# Influenza A virus M2 protein triggers mitochondrial DNA-mediated antiviral immune responses

**DOI:** 10.1038/s41467-019-12632-5

**Published:** 2019-10-11

**Authors:** Miyu Moriyama, Takumi Koshiba, Takeshi Ichinohe

**Affiliations:** 1 Division of Viral Infection, Department of Infectious Disease Control, International Research Center for Infectious Diseases, Institute of Medical Science, The University of Tokyo, Minato-ku, Tokyo 108-8639 Japan; 2 Department of Chemistry, Faculty of Science, Fukuoka University, Jonan-ku, Fukuoka 814-0180 Japan; 3 Present Address: Department of Immunobiology, Yale University School of Medicine, New Haven, CT 06519 USA

**Keywords:** Pattern recognition receptors, Influenza virus, Restriction factors, Viral host response

## Abstract

Cytosolic mitochondrial DNA (mtDNA) activates cGAS-mediated antiviral immune responses, but the mechanism by which RNA viruses stimulate mtDNA release remains unknown. Here we show that viroporin activity of influenza virus M2 or encephalomyocarditis virus (EMCV) 2B protein triggers translocation of mtDNA into the cytosol in a MAVS-dependent manner. Although influenza virus-induced cytosolic mtDNA stimulates cGAS- and DDX41-dependent innate immune responses, the nonstructural protein 1 (NS1) of influenza virus associates with mtDNA to evade the STING-dependent antiviral immunity. The STING-dependent antiviral signaling is amplified in neighboring cells through gap junctions. In addition, we find that STING-dependent recognition of influenza virus is essential for limiting virus replication in vivo. Our results show a mechanism by which influenza virus stimulates mtDNA release and highlight the importance of DNA sensing pathway in limiting influenza virus replication.

## Abstract (web summary)

Cytosolic mitochondrial DNA (mtDNA) plays a role in innate antiviral immunity but how this is triggered during infection remains unclear. Here, the authors provide evidence that the Influenza virus protein M2 stimulates translocation of mtDNA into the cytosol in a MAVS-dependent manner.

## Introduction

The detection of viral nucleic acids is a major strategy by which the innate immune system senses viral infection^1,2^. Cyclic guanosine monophosphate-adenosine monophosphate (cGAMP) synthase (cGAS, also known as MB21D1) detects double-stranded DNA (dsDNA) derived from DNA viruses such as herpes simplex virus 1 (HSV-1) and vaccinia virus^3–5^ or reverse-transcribed DNA of retroviruses^6^. In the presence of dsDNA, cGAS produces cGAMP, which in turn binds to and activates stimulator of interferon genes (STING, also known as MITA, MPYS, ERIS, and TMEM173) that resides in the endoplasmic reticulum (ER) to enhance interferon (IFN)-β expression and innate immune defense against HSV-1 infection^7–11^. The cGAS-produced cGAMP is transferred from vaccinia virus-infected cells to adjacent non-infected cells through gap junction to facilitate STING-dependent antiviral immune signaling^12^. In addition, recent reports indicate that certain enveloped DNA viruses, including vaccinia virus and murine cytomegalovirus, HSV-1, and retroviruses incorporate and transfer cGAMP to newly infected cells to trigger STING-dependent IFN responses^13,14^.

Interestingly, STING also plays a critical role in production of IFN-β and limiting viral replication after infection with certain RNA viruses, such as vesicular stomatitis virus (VSV)^7^, Sendai virus (SeV)^7,9,11^, EMCV^7^, or Newcastle disease virus (NDV)^11^. It has been demonstrated that membrane fusion activity of influenza virus stimulates interferon production in a STING-dependent manner^15^. In addition, STING inhibits the translation of viral mRNAs to prevent viral protein synthesis during VSV infection^16^. Furthermore, recent studies highlighted the importance of cytosolic mtDNA in cGAS-mediated antiviral immune responses after infection with certain RNA viruses, such as VSV, lymphocytic choriomeningitis virus (LCMV), Sindbis virus, and dengue virus^17–20^. Accumulation of cytosolic mtDNA induced by TFAM (transcription factor A, mitochondrial) deficiency promotes the cGAS-dependent interferon-stimulated genes (ISGs) expression and confers resistance to acute infection by LCMV^18^. In addition, Aguirre et al. showed that dengue virus NS2B protein degrades cGAS to prevent its activation by cytosolic mtDNA released during dengue virus infection^17^. Although these observations indicate that cytosolic mtDNA promotes cGAS-STING-dependent innate antiviral immunity and confers resistance to RNA viruses^17–20^, the mechanism by which RNA virus infection stimulates mtDNA release remains unclear.

In this study, we examine the cellular mechanism by which influenza virus infection triggers mtDNA release. We demonstrate that viroporin activity of influenza virus M2 or EMCV 2B protein is essential for cytosolic mtDNA release into the cytosol. In addition, influenza virus NS1 protein associates with cytosolic mtDNA to attenuate cGAS- and DDX41-dependent innate immune responses. Furthermore, we find that the STING-dependent signals are essential for limiting influenza virus replication in vivo. These results highlight the importance of cytosolic mtDNA in influenza virus-induced innate antiviral immune responses.

## Results

### Influenza A virus triggers mtDNA release

**Fig. 1:**
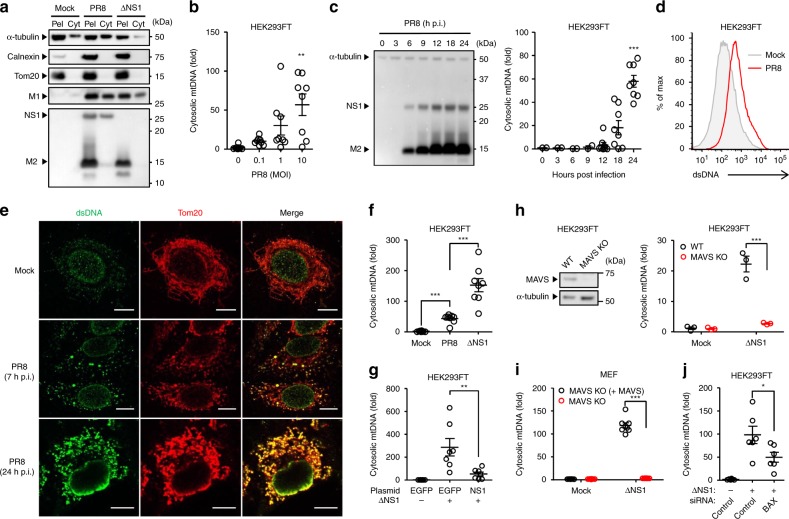
Influenza virus triggers mtDNA release. **a** HEK293FT cells were subjected to digitonin fractionation as described in the Methods and pellets (Pel) or cytosolic extracts (Cyt) were analyzed by western blotting using the indicated antibodies. **b** HEK293FT cells were infected with PR8 virus at indicated MOIs. Cytosolic mtDNA was assessed by quantitative PCR. **c** HEK293FT cells were infected with PR8 virus at MOI of 10. Cell lysates were collected at indicated time points and analyzed by immunoblotting with indicated antibodies (left panel). Cytosolic mtDNA was assessed by quantitative PCR (right panel). **d** HEK293FT were infected with PR8 virus. Cells were collected at 24 h post infection, and intracellularly stained with dsDNA-specific antibody (35I9 DNA). **e** STING-A549 cells were infected with PR8 virus. At indicated time points, cells were stained with anti-dsDNA (AC-30-10) and anti-Tom20 antibodies and analyzed by confocal microscopy. Scale bars, 10 μm. **f** HEK293FT cells were infected with WT or ΔNS1 influenza virus at MOI of 10. Cytosolic mtDNA was assessed by quantitative PCR. **g** HEK293FT cells were transfected with the expression plasmid encoding EGFP or Flag-tagged NS1 protein. Twenty-four hours after transfection, the cells were infected with ΔNS1 influenza virus for 24 h. Cytosolic mtDNA was assessed by quantitative PCR. **h** WT or MAVS KO HEK293FT cells were infected with ΔNS1 influenza virus for 24 h. Cell lysates were collected and analyzed by immunoblot using indicated antibodies (left panel). Cytosolic mtDNA was assessed by quantitative PCR (right panel). **i** MAVS KO MEFs, or MAVS KO MEFs re-introduced full-length MAVS (MAVS KO (+MAVS)) were infected with ΔNS1 influenza virus for 24 h. Cytosolic mtDNA was assessed by quantitative PCR. **j** HEK293FT cells transfected with siRNA targeting *Bax* or control siRNA were infected with ΔNS1 influenza virus for 24 h. Cytosolic mtDNA was assessed by quantitative PCR. These data are from three independent experiments (**b**, **c**, **f**–**j**; mean ± s.e.m.). *P < 0.05, **P < 0.01, ***P < 0.001; (one-way ANOVA and Tukey’s test). Source data are provided as a Source Data file

Infection with HSV-1, HSV-2, and murine gammaherpervirus 68 (MHV-68) but not VSV, influenza, LCMV, or vaccinia virus triggers mtDNA stress in mouse embryonic fibroblasts (MEFs)^18^. In addition, dengue virus stimulates mtDNA release into the cytosol in human lung carcinoma cell line A549^17,19^. To determine whether influenza virus stimulates mtDNA release in a cell type-specific manner, we first examined mtDNA release in candidate cell types, including mouse lung fibroblasts, human A549, and human embryonic kidney cell line 293FT (HEK293FT) cells, in which influenza virus infected and replicated efficiently (Supplementary Fig. 1). To detect mtDNA in the cytosol, we first extracted pure cytosolic fractions from mock- or virus-infected cells (Fig. 1a). Analysis of the pure cytosolic extracts demonstrated that wild-type (WT) influenza A virus significantly induced mtDNA release in HEK293FT cells (Fig. 1b, c), A549 cells stably expressing the STING protein (termed STING-A549 cells), and mouse lung fibroblasts (Supplementary Fig. 2) in a multiplicity of infection (MOI)-dependent manner within 24 h post infection. Flow cytometric and confocal microscopic analysis confirmed that influenza virus-induced high levels of dsDNA in cytosol (Fig. 1d, e). In addition, we found that cytosolic mtDNA release was significantly enhanced by infection with recombinant influenza virus lacking the NS1 gene (ΔNS1), which is known to induce large amounts of type I IFNs^21^ and commonly used as a potent IFN inducer, compared with that of WT virus (Fig. 1f and Supplementary Fig. 2d, h). The NS1 protein of influenza virus inhibits IFN responses including IFN regulatory factor 3 (IRF3) phosphorylation either by sequestering viral RNA or binding to RIG-I and other host proteins required for RIG-I- and MAVS-dependent signaling pathways^22–26^. These observations suggest that the NS1 protein of influenza virus directly inhibits mtDNA release into the cytosol or full activation of RIG-I/MAVS-dependent signals are essential for mtDNA release in influenza virus-infected cells. To address these possibilities, we infected EGFP- or NS1-transfected HEK293FT cells with ΔNS1 influenza virus. The cytosolic translocation of mtDNA in response to ΔNS1 influenza virus was significantly inhibited by the NS1 protein (Fig. 1g). We next examined whether MAVS is required for influenza virus-induced mtDNA release. To this end, we infected WT or MAVS-deficient HEK293FT cells with ΔNS1 influenza virus. Strikingly, we found that influenza virus-induced mtDNA release into the cytosol was diminished in MAVS-deficient HEK293FT cells (Fig. 1h). To provide definitive evidence that MAVS was responsible for influenza virus-induced mtDNA release, we performed a complementation experiment. Intact MAVS expressed from a lentivirus was able to completely restore the ability of MAVS-deficient MEFs to trigger cytosolic mtDNA release after ΔNS1 influenza virus infection (Fig. 1i). Previous studies have demonstrated that Bax/Bak play a critical role for mtDNA release into the cytosol^27,28^. In the case of SeV infection, the virus activated-IRF3 associates with Bax to translocate to the mitochondria and cause cytochrome c release^29^. Indeed, infection with ΔNS1 influenza virus induced more IRF3 phosphorylation than the WT virus (Supplementary Fig. 3a). In addition, both WT and ΔNS1 influenza virus-induced IRF3 phosphorylation in a MAVS-dependent manner (Supplementary Fig. 3b). Furthermore, the cytosolic mtDNA release was significantly reduced in BAX knockdown HEK293FT cells after ΔNS1 influenza virus infection (Fig. 1j). Together, these data suggest that influenza virus stimulates mtDNA release into the cytosol and MAVS-dependent signals are required for mtDNA release upon influenza virus infection.

### Influenza virus M2 protein triggers cytosolic mtDNA release

**Fig. 2:**
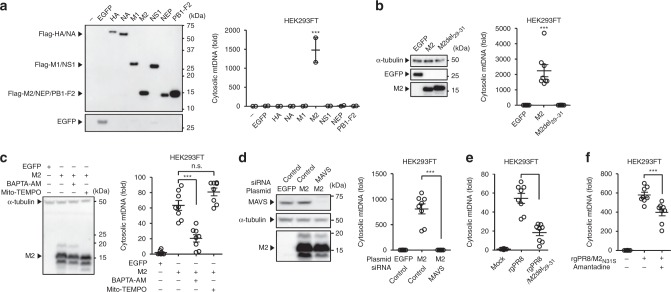
Ion channel activity of influenza virus M2 protein is essential for mtDNA release. **a** HEK293FT cells were transfected with the expression plasmid encoding EGFP or Flag-tagged influenza virus proteins. Cell lysates were collected at 24 h post transfection and analyzed by immunoblot with mouse monoclonal antibody against Flag or EGFP (left panel). Cytosolic mtDNA was assessed by quantitative PCR at 24 h post transfection (right panel). **b** HEK293FT cells were transfected with the expression plasmid encoding EGFP or Flag-tagged WT or mutant M2 protein. Cell lysates were collected at 24 h post transfection and analyzed by immunoblot using indicated antibodies (left panel). Cytosolic mtDNA was assessed by quantitative PCR at 24 h post transfection (right panel). **c** HEK293FT cells were transfected with the expression plasmid encoding EGFP or Flag-tagged M2 protein in the presence or absence of BAPTA-AM (20 μM) or Mito-TEMPO (500 μM). Cell lysates were collected at 24 h post transfection and blotted using the indicated antibodies (left panel). Cytosolic mtDNA was assessed by quantitative PCR at 24 h post transfection (right panel). **d** HEK293FT cells were transfected with siRNA targeting *MAVS* or control siRNA. Two days later, cells were transfected with the expression plasmid encoding EGFP or Flag-tagged M2 protein. Cell lysates were collected at 24 h post DNA transfection and blotted using the indicated antibodies (left panel). Cytosolic mtDNA was assessed by quantitative PCR at 24 h post DNA transfection (right panel). **e**, **f** HEK293FT cells were infected with WT (rgPR8), M2del29–31 virus (rgPR8/M2del29–31) (**e**), or amantadine sensitive-recombinant influenza virus (rgPR8/M2_N31S_) in the presence or absence of amantadine (100 μM) (**f**). Cytosolic mtDNA was assessed by quantitative PCR at 24 h post infection. These data are from three independent experiments (**a**–**f**; mean ± s.e.m.). ***P < 0.001; n.s., not significant (one-way ANOVA and Tukey’s test). Source data are provided as a Source Data file

We next examined the mechanism by which influenza virus stimulates mtDNA release. To identify influenza virus protein that may stimulate mtDNA release into the cytosol, we transfected HEK293FT cells with expression plasmids encoding influenza virus protein. Remarkably, we found that M2 protein of influenza virus was sufficient to trigger mtDNA release in transfected HEK293FT cells (Fig. 2a). Influenza virus M2 protein, a proton-selective ion channel, is important for the viral uncoating during entry and virus budding^30^. To test whether M2 ion-channel activity is needed to elicit mtDNA release, we transfected HEK293FT cells with the expression plasmid encoding influenza virus M2 protein lacking amino acids 29–31 from the transmembrane region (M2del_29–31_). These amino acids are required for M2 ion channel activity^31^. Notably, mtDNA release was completely absent in cells transfected with M2del29–31 plasmid (Fig. 2b). Treatment of cells with the cell-permeable Ca^2+^-chelator BAPTA-AM but not antioxidant Mito-TEMPO, a scavenger specific for mitochondrial ROS, significantly blocked cytosolic mtDNA release by influenza virus M2 protein without affecting the expression levels of the M2 protein (Fig. 2c). In addition, the M2-induced cytosolic mtDNA release was MAVS dependent (Fig. 2d). We next examined whether the requirement of M2 ion channel activity for mtDNA release can be reproduced by infection with recombinant influenza virus lacking amino acids 29–31 of the M2 protein. To this end, we generated recombinant influenza virus lacking amino acids 29–31 of the M2 protein (rgPR8/M2del_29–31_ virus) (Supplementary Fig. 4a). Consistent with our previous report^32^, the rgPR8/M2del_29–31_ virus failed to stimulate the release of IL-1β from LPS-primed bone marrow-derived dendritic cells (BMDCs) or macrophages (BMMs) (Supplementary Fig. 4b, c). Although, the extent of infection by the rgPR8/M2del_29–31_ virus in HEK293FT cells was similar to that of WT rgPR8 virus (Supplementary Fig. 4d, e), the M2del_29–31_ virus significantly reduced cytosolic release of mtDNA from HEK293FT cells compared to the WT virus-infected cells (Fig. 2e). In addition, amantadine sensitive-recombinant influenza virus (rgPR8/M2_N31S_)^33^ significantly reduced cytosolic release of mtDNA from HEK293FT cells in the presence of amantadine (Fig. 2f). We previously demonstrated that ion channel activity of the influenza virus M2 protein is essential for caspase-1 activation^32^. In addition, previous studies have demonstrated that caspase-1- or caspase-11-cleaved GSDMD induces the formation of a plasma membrane pore^34–36^. These observations prompted us to examine whether GSDMD is involved in mtDNA release from the mitochondria upon influenza virus infection. Consistent with previous results^35^, overexpression of GSDMD residues 1–275 (GSDMD_1–275_) in HEK293FT cells results in cell death (Supplementary Fig. 5a). However, we found that GSDMD_1–275_ expression alone was insufficient to trigger mtDNA release from mitochondria (Supplementary Fig. 5b). In addition, HEK293FT cells, in which the influenza virus stimulated mtDNA release into the cytosol, did not express detectable levels of GSDMD (Supplementary Fig. 5c). Collectively, these data indicate that the ion-channel activity of M2 protein is important for mtDNA release in influenza virus-infected cells.

### EMCV viroporin 2B stimulates mtDNA release

**Fig. 3:**
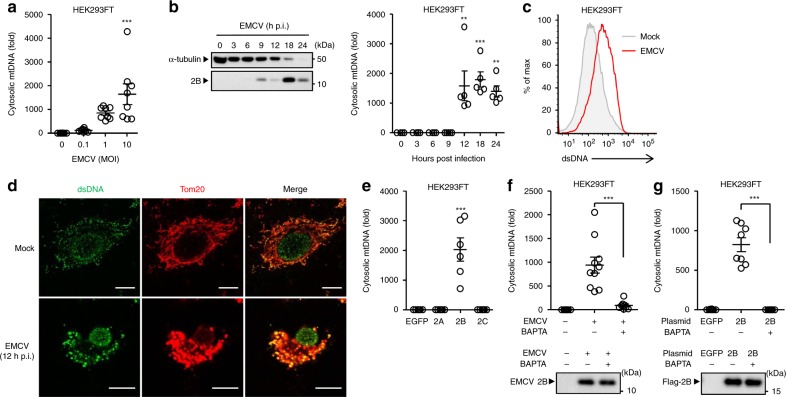
EMCV 2B protein stimulates cytosolic mtDNA release. **a** HEK293FT cells were infected with EMCV at indicated MOIs. Cytosolic mtDNA was assessed by quantitative PCR. **b** HEK293FT cells were infected with EMCV virus at MOI of 10. Cell lysates were collected at indicated time points and analyzed by immunoblotting with indicated antibodies (left panel). Cytosolic mtDNA was assessed by quantitative PCR (right panel). **c** HEK293FT were infected with EMCV. Cells were collected at 24 h post infection, and intracellularly stained with dsDNA-specific antibody (35I9 DNA). **d** STING-A549 cells were infected with EMCV. At 12 h post infection, cells were stained with anti-dsDNA (AC-30-10) and anti-Tom20 antibodies and analyzed by confocal microscopy. Scale bars, 10 μm. **e** HEK293FT cells were transfected with the expression plasmid encoding EGFP or Flag-EMCV 2A, 2B, or 2C protein. Cytosolic mtDNA was assessed by quantitative PCR at 24 h post transfection. **f**, **g** HEK293FT cells were infected with EMCV (**f**) or transfected with the expression plasmid encoding EGFP or Flag-tagged 2B protein (**g**) in the presence or absence of BAPTA (20 μM). Cell lysates were collected at 18 h post infection (**f**) or transfection (**g**) and analyzed by immunoblot with rabbit polyclonal antibody against EMCV 2B protein (**f**, lower panel) or mouse monoclonal antibody against Flag (**g**, lower panel). Cytosolic mtDNA was assessed by quantitative PCR at 18 h post infection (**f**, upper panel) or transfection (**g**, upper panel). These data are from three independent experiments (**a**, **b**, **e**–**g**; mean ± s.e.m.). **P < 0.01, ***P < 0.001; (one-way ANOVA and Tukey’s test). Source data are provided as a Source Data file

Next, we wished to determine whether the viroporins, transmembrane pore-forming viral proteins, of other RNA viruses could stimulate release of mtDNA into the cytosol. Infection with EMCV significantly stimulated mtDNA release in HEK293FT cells in a MOI-dependent manner within 24 h post infection (Fig. 3a, b and Supplementary Fig. 1a). Flow cytometric and confocal microscopic analysis confirmed that EMCV induced high levels of dsDNA in cytosol (Fig. 3c, d). We have previously demonstrated that EMCV viroporin 2B activates the NLRP3 inflammasome by stimulating Ca^2+^ flux from intracellular storages to the cytosol^37^. Thus, we next examined whether 2B protein of EMCV is sufficient to trigger mtDNA release into the cytosol. Notably, overexpression of the 2B protein in HEK293FT cells significantly increased mtDNA release into the cytosol (Fig. 3e). In addition, treatment of HEK293FT cells with the BAPTA-AM blocked mtDNA release by EMCV infection or overexpression of 2B protein without affecting the expression levels of the 2B protein (Fig. 3f, g). Taken together, these results suggest that viroporin activity of influenza virus and EMCV are essential for mtDNA release.

### Influenza virus stimulates cGAS- and DDX41-dependent IFN-β mRNA

**Fig. 4:**
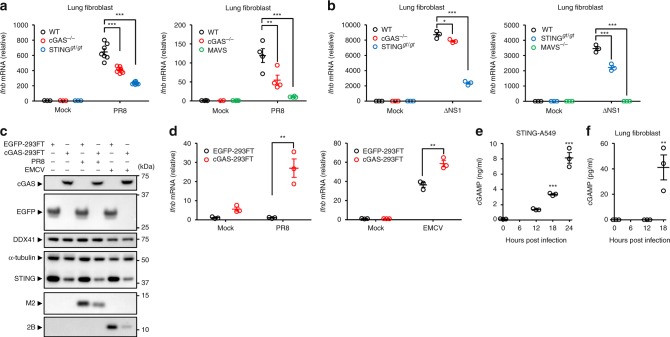
Influenza virus stimulates cGAS- and STING-dependent IFN-β gene expression in mouse lung fibroblast. **a**, **b** Primary lung fibroblast prepared from WT, cGAS-, STING-, and MAVS-deficient mice were infected with WT PR8 (**a**) or ΔNS1 influenza virus (**b**). IFN-β mRNA levels were assessed by quantitative PCR with GAPDH as an internal control. **c** Samples from HEK293FT cells stably expressing EGFP (EGFP-293FT) or cGAS (cGAS-293FT) were infected with PR8 or EMCV. Cell lysates were collected at 9 h post infection and blotted using the indicated antibodies. **d** EGFP-293FT or cGAS-293FT cells were infected with influenza virus (left panel) or EMCV (right panel) for 24 h. IFN-β mRNA levels were assessed by quantitative PCR with β-actin as an internal control. **e**, **f** STING-A549 cells (**e**) or lung fibroblasts (**f**) were infected with PR8 virus. Pure cytosolic extracts were collected at indicated time points and analyzed for cGAMP by ELISA. These data are from three independent experiments (**a**, **b**, **d**–**f**; mean ± s.e.m.). *P < 0.05, **P < 0.01, ***P < 0.001; (one-way ANOVA and Tukey’s test). Source data are provided as a Source Data file

Given that mtDNA triggers cGAS-dependent innate immune responses^18,19,27,28^, the cytosolic mtDNA in influenza virus- or EMCV-infected cells may trigger cGAS-dependent antiviral immune responses. To test this idea, we infected cGAS-, STING-, or MAVS-deficient primary lung fibroblasts with influenza virus. Adenovirus-induced IFN-β gene expression was largely dependent on cGAS and STING (Supplementary Fig. 6). Notably, the expression of IFN-β gene was significantly reduced in cGAS-, STING-, and MAVS-deficient cells upon influenza virus or EMCV infection (Fig. 4a, b and Supplementary Figs. 7 and 8). Since HEK293FT cells did not express detectable levels of the cGAS protein, we established HEK293FT cells stably expressing EGFP or the cGAS protein termed EGFP-293FT or cGAS-293FT cells, respectively. Although overexpression of cGAS reduced the expression levels of STING (Supplementary Fig. 9), we could detect the STING in cGAS-293FT cells (Fig. 4c). Importantly, the cGAS-293FT cells significantly potentiated IFN-β gene expression after influenza virus or EMCV infection (Fig. 4d). In addition, influenza virus-induced high levels of cGAMP in STING-A549 cells or primary lung fibroblasts within 24 h post infection (Fig. 4e, f). Since TRIM32-mediated K63-linked ubiquitination of STING is important for SeV or HSV-1- induced antiviral immune responses^38^, we established TRIM32-knockout STING-A549 cells using the CRISPR/Cas9 system (Supplementary Fig. 10a) and found that TRIM32-knockout STING-A549 cells significantly reduced the IFN-β gene expression after influenza virus infection (Supplementary Fig. 10b). These data indicate that cGAS-dependent signaling could be activated upon influenza virus infection.

**Fig. 5:**
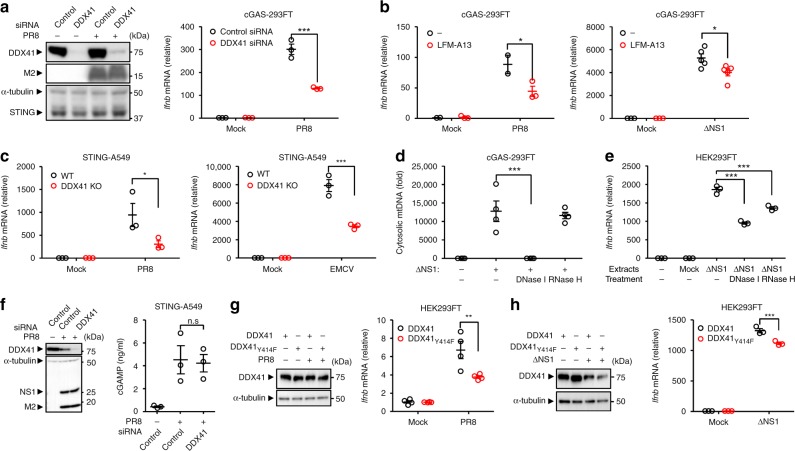
Influenza virus stimulates DDX41-dependent IFN-β gene expression. **a** cGAS-293FT cells transfected with siRNA targeting *DDX41* or control siRNA were infected with influenza virus for 24 h. Cell lysates were collected and blotted using the indicated antibodies (left panel). IFN-β mRNA levels were assessed by quantitative PCR with β-actin as an internal control (right panel). **b**, **c** cGAS-293FT cells were infected with WT (left panel) or ΔNS1 influenza virus (right panel) for 24 h in the presence or absence of LFM-A13 (100 μM) (**b**). WT or DDX41-deficient STING-A549 cells were infected with PR8 (left panel), or EMCV (right panel) for 24 h (**c**). IFN-β mRNA levels were assessed by quantitative PCR with β-actin as an internal control. **d** Pure cytosolic fraction prepared from digitonin extracts of mock- or ΔNS1 influenza virus-infected cGAS-293FT cells were treated with DNase I or RNase H. Cytosolic mtDNA was assessed by quantitative PCR. **e** HEK293FT cells were transfected with DNA extracted from DNase I- or RNase H-treated pure cytosolic fraction for 24 h. IFN-β mRNA levels were assessed by quantitative PCR with β-actin as an internal control. **f** STING-A549 cells transfected with siRNA targeting *DDX41* or control siRNA were infected with PR8 virus for 24 h. Cell lysates were collected at 24 h post infection and blotted using the indicated antibodies (left panel). Pure cytosolic extracts were collected at 24 h post infection and analyzed for cGAMP by ELISA (right panel). **g**, **h** HEK293FT cells were transfected with siRNA targeting *DDX41*. Two days later, cells were transfected with the expression plasmid encoding Flag-tagged DDX41 or DDX41 (Y414F) mutant. Twenty-four hours after DNA transfection, the cells were infected with WT (**g**) ΔNS1 influenza virus (**h**) for 24 h. Cell lysates were collected at 24 h post infection and blotted using the indicated antibodies (left panel). IFN-β mRNA levels were assessed by quantitative PCR with β-actin as an internal control (right panel). These data are from three independent experiments (mean ± s.e.m.). **P < 0.01, ***P < 0.001; n.s., not significant (one-way ANOVA and Tukey’s test). Source data are provided as a Source Data file

Although HEK293FT cells did not express detectable levels of the cGAS protein (Fig. 4c and Supplementary Fig. 9), we found that the ΔNS1 influenza virus-induced IFN-β gene expression was dependent on STING in HEK293FT cells (Supplementary Fig. 11a, b). This led us to consider the possibility that other DNA sensors may play a role in the induction of IFN-β gene expression after influenza virus infection. In the case of retrovirus infection, DDX41 recognizes RNA/DNA hybrid reverse transcription intermediates^39^. In addition, mtDNA contains RNAs hybridized to DNA^40^. These observations prompted us to examine whether influenza virus-induced cytosolic mtDNA may trigger DDX41-dependent innate immune response. To address this possibility, we treated HEK293FT cells with siRNA targeting *DDX41* (Supplementary Table 1). Although knockdown of DDX41 in D2SC cells, a mouse myeloid DC line, has no effect on influenza virus-induced IFN-α/β production^41^, we found that IFN-β gene expression was significantly reduced in DDX41 knockdown HEK293FT or cGAS-293FT cells after infection with WT or ΔNS1 influenza virus (Fig. 5a and Supplementary Fig. 11c, d). In addition, inhibition of Bruton’s tyrosine kinase (BTK), which phosphorylates DDX41 to facilitate STING-dependent induction of IFN-β gene expression^42^, by a chemical inhibitor LFM-A13 significantly reduced influenza virus-induced IFN-β gene expression in cGAS-293FT cells (Fig. 5b**)**. To confirm the importance of DDX41 in influenza virus-induced IFN-β gene expression, we established DDX41-knockout STING-A549 cells using the CRISPR/Cas9 system (Supplementary Fig. 12a). Although DDX41-knockout STING-A549 cells released comparable levels of mtDNA into the cytosol upon influenza virus infection (Supplementary Fig. 12b), DDX41-knockout STING-A549 cells significantly reduced the IFN-β gene expression after infection with influenza virus or EMCV (Fig. 5c). In the case of retrovirus infection, DDX41 recognizes RNA/DNA hybrid reverse transcription intermediates^39^. Thus, we next tested the effects of ribonuclease H (RNase H), an endoribonuclease which specifically degrades the RNA strand of an RNA/DNA hybrid, on IFN-β gene expression after influenza virus infection. Although treatment of pure cytosolic extracts of ΔNS1 influenza virus-infected cells with RNase H did not change the levels of cytosolic mtDNA (Fig. 5d), transfection with RNase H-treated cytosolic extracts significantly reduced IFN-β gene expression in HEK293FT cells (Fig. 5e), suggesting that RNA/DNA hybrid could play an important role in influenza virus-induced IFN-β gene expression. Although treatment of STING-A549 cells with siRNA targeting *DDX41* did not affect the levels of cGAMP following influenza virus infection (Fig. 5f), mutation of Tyr414, which is critical for recruitment of STING to DDX41^42^, to phenylalanine (Y414F) inhibited the IFN-β gene expression (Fig. 5g, h). Together, these data suggest that DDX41 is important for influenza virus-induced IFN-β gene expression.

We further examined the importance of mtDNA in influenza virus-induced IFN-β gene expression. To this end, we generated mtDNA-depleted ρ^0^ HEK293FT (Supplementary Fig. 13a). The ρ^0^ HEK293FT cells abrogated mtDNA release into the cytosol in response to ΔNS1 influenza virus infection (Supplementary Fig. 13b). Although depletion of mtDNA did not change the expression levels of DDX41, RIG-I, MAVS, and STING (Supplementary Fig. 13c), ρ^0^ HEK293FT cells significantly reduced the IFN-β gene expression after infection with ΔNS1 influenza virus (Supplementary Fig. 13d). Similarly, ρ^0^ cGAS-293FT cells significantly reduced the IFN-β gene expression after infection with WT or ΔNS1 influenza virus (Supplementary Fig. 14), suggesting that mtDNA is important for influenza virus-induced IFN-β gene expression. Collectively these data indicated that influenza virus infection stimulates cGAS- and DDX41-dependent IFN-β gene expression in a cell type-specific manner.

### Influenza virus NS1 protein associates with mtDNA

**Fig. 6:**
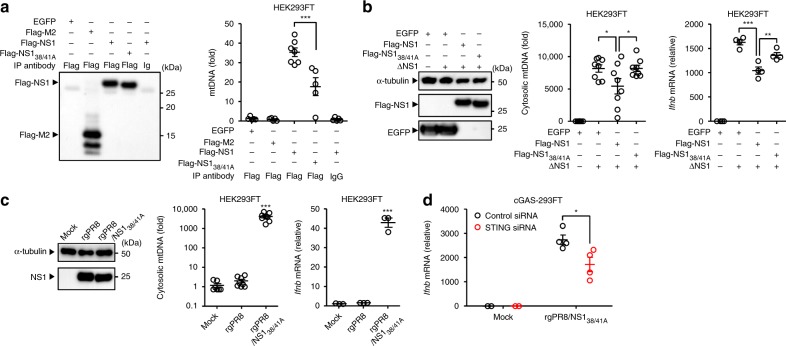
Influenza virus NS1 protein associates with mtDNA. **a** HEK293FT cells were transfected with the expression plasmid encoding EGFP, Flag-tagged M2, NS1, or NS1_38/41_ mutant. Twenty-four hours after transfection, the cells were infected with ΔNS1 influenza virus for 24 h. Pure cytosolic extracts prepared from digitonin extracts of ΔNS1 influenza virus-infected cells were immunoprecipitated with mouse monoclonal antibody against Flag, followed by immunoblotting of immunoprecipitates with rabbit polyclonal antibody against Flag (left panel). DNA was extracted from immunoprecipitated samples using QIAquick Nucleotide Removal kit (QIAGEN). NS1-bound mtDNA was assessed by quantitative PCR (right panel). **b** HEK293FT cells were transfected with the expression plasmid encoding EGFP, Flag-tagged NS1, or NS1 _38/41_ mutant. Twenty-four hours after transfection, the cells were infected with ΔNS1 influenza virus for 24 h. Cell lysates were collected and blotted using the indicated antibodies (left panel). Cytosolic mtDNA was assessed by quantitative PCR (middle panel). IFN-β mRNA levels were assessed by quantitative PCR with β-actin as an internal control (right panel). **c** HEK293FT cells were infected with WT (rgPR8) or rgPR8/NS1_38/41A_ influenza virus at MOI of 1 for 24 h. Cell lysates were collected and blotted using the indicated antibodies (left panel). Cytosolic mtDNA (middle panel) and IFN-β mRNA levels (right panel) were assessed by quantitative PCR. **d** cGAS-293FT cells transfected with siRNA targeting *STING* or control siRNA were infected with rgPR8/NS1_38/41A_ influenza virus for 24 h. IFN-β mRNA levels were assessed by quantitative PCR with β-actin as an internal control. These data are from three independent experiments (mean ± s.e.m.). *P < 0.05, **P < 0.01, ***P < 0.001; (one-way ANOVA and Tukey’s test). Source data are provided as a Source Data file

The NS1 protein of influenza virus binds dsRNA with no sequence specificity to prevent dsRNA-activated protein kinase (PKR)-mediated antiviral responses^43–47^. In addition, it has been reported that influenza virus NS1 protein binds cellular dsDNA to inhibit the transcription of antiviral genes^48^. This led us to consider the possibility that NS1 protein of influenza virus may associate with cytosolic mtDNA to inhibit cGAS- or DDX41-dependent innate immune responses. To address this possibility, we first performed subcellular fractionation and immunoprecipitation studies. Flag-tagged NS1 or M2 proteins were immunoprecipitated by anti-flag or control antibodies in HEK293FT cells after infection with ΔNS1 influenza virus. Although the NS1 protein of influenza virus did not co-localize with dsDNA in cytosol of influenza virus-infected cells (Supplementary Fig. 15), we found that the flag-tagged NS1 protein specifically coimmunoprecipitated with cytosolic mtDNA after infection with ΔNS1 influenza virus (Fig. 6a and Supplementary Fig. 16). Similarly, influenza virus NS1 protein was coimmunoprecipitated with an antibody against dsDNA (Supplementary Fig. 17a, b). In addition, the anti-dsDNA antibody coimmunoprecipitated with cGAS, DDX41, and TFAM in cGAS-293FT cells after infection with WT PR8 influenza virus (Supplementary Fig. 17b). Further, the biotin-tagged 45 bp interferon stimulatory DNA (ISD) coimmunoprecipitated with NS1 and DDX41 in cGAS-293FT cells after infection with WT PR8 influenza virus (Supplementary Fig. 18). Since two basic amino acids at positions 38 and 41 within the NS1 protein are known to be important for its dsRNA- or dsDNA-binding activity^45,48,49^, we next examined the role of these two amino acids (R38 and K41) in association between the NS1 protein and mtDNA and found that the flag-tagged R38A/K41A NS1 mutant reduced association with cytosolic mtDNA after infection with ΔNS1 influenza virus (Fig. 6a). Consequently, the R38A/K41A NS1 mutant failed to inhibit cytosolic translocation of mtDNA and IFN-β gene expression after ΔNS1 influenza virus infection (Fig. 6b). In addition, infection with a recombinant influenza virus expressing the mutant NS1 R38A/K41A protein (rgPR8/NS1_38/41A_) significantly enhanced the detectable levels of cytosolic mtDNA and STING-dependent IFN-β gene expression compared to WT virus-infected cells without affecting the expression levels of the NS1 protein (Fig. 6c, d). Collectively these data indicated that influenza virus NS1 protein may associate with mtDNA through its RNA-binding domain to block STING-dependent IFN-β gene expression.

### NS1 protein mitigate immunostimulatory potential of mtDNA

**Fig. 7:**
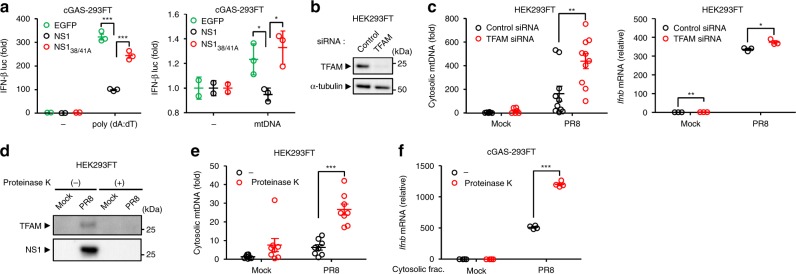
NS1 binding to mtDNA attenuates its immunostimulatory potential. **a** cGAS-293FT cells co-transfected with expression plasmids encoding EGFP, Flag-tagged NS1, or NS1_38/41_ mutant, together with IFN-β reporter plasmids and poly(dA:dT) (left panel) or mtDNA (right panel). Twenty-four hours after transfection, cell lysates were collected and analyzed for luciferase activity. **b** Samples from HEK293FT cells transfected with siRNA targeting *TFAM* or control siRNA were blotted using the indicated antibodies. **c** HEK293FT cells transfected with siRNA targeting *TFAM* or control siRNA were infected with PR8 virus for 24 h. Cytosolic mtDNA (left panel) and IFN-β mRNA levels (right panel) were assessed by quantitative PCR. **d** Pure cytosolic fraction prepared from digitonin extracts of mock- or PR8-infected HEK293FT cells were treated with proteinase K. Proteinase K-treated pure cytosolic extracts were analyzed by immunoblotting with indicated antibodies. **e** DNA was extracted from proteinase K-treated pure cytosolic fraction using QIAquick Nucleotide Removal kit (QIAGEN). Cytosolic mtDNA was assessed by quantitative PCR. **f** cGAS-293FT cells were transfected with DNA extracted from proteinase K-treated pure cytosolic fraction for 6 h. IFN-β mRNA levels were assessed by quantitative PCR with β-actin as an internal control. These data are from three independent experiments (**a**, **c**, **e**, **f**; mean ± s.e.m.). *P < 0.05, **P < 0.01, ***P < 0.001; (one-way ANOVA and Tukey’s test). Source data are provided as a Source Data file

Since the NS1 protein of influenza virus associated with mtDNA, we next examined whether the NS1 protein inhibits dsDNA- or mtDNA-mediated activation of IFN-β promoter activity. WT but not R38A/K41A NS1 protein significantly inhibited dsDNA- or mtDNA-mediated activation of IFN-β promoter activity (Fig. 7a). This led us to consider the possibility that depletion of the mtDNA-associated proteins including NS1 or TFAM may enhance the ability of mtDNA to stimulate innate immune responses. Consistent with a previous report^18^, TFAM depletion enhanced detectable levels of cytosolic mtDNA and basal expression levels of IFN-β at steady state (Fig. 7b, c). In addition, TFAM depletion significantly enhanced detectable levels of cytosolic mtDNA and IFN-β gene expression after influenza virus infection (Fig. 7c). Further, treatment of pure cytosolic extracts of influenza virus-infected cells with proteinase K depleted both TFAM and NS1 proteins (Fig. 7d) and enhanced detectable levels of cytosolic mtDNA after influenza virus infection (Fig. 7e and Supplementary Fig. 19). Consequently, transfection of cGAS-293FT cells with proteinase K-treated cytosolic extracts from influenza virus-infected cells significantly enhanced IFN-β gene expression compared with untreated control extracts of influenza virus-infected cells (Fig. 7f). In addition, we found that infection with EMCV but not influenza virus or adenovirus reduced the expression levels of TFAM (Supplementary Fig. 20). Collectively, these results indicate that the NS1 protein of influenza virus may associate with mtDNA to evade recognition by cytosolic DNA sensors.

### Connexin 43 amplifies STING-dependent antiviral response

**Fig. 8:**
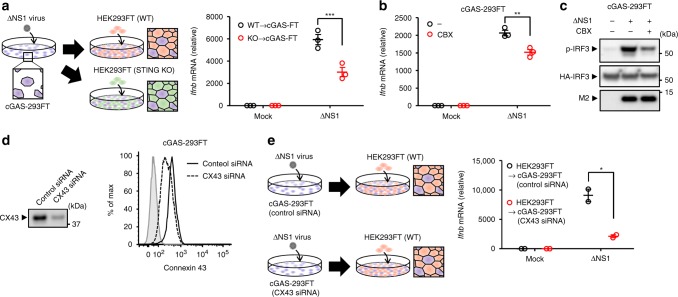
Connexin 43 amplifies influenza virus-induced STING-dependent innate immune signaling. **a** Schematic representation of experimental setup (left panel). cGAS-293FT cells were infected with ΔNS1 influenza virus (purple). Six hours later, uninfected WT (red) or STING KO (green) HEK293FT cells were added to the ΔNS1 influenza virus-infected cGAS-293FT cells (purple) and co-cultured for additional 18 h. IFN-β mRNA levels were assessed by quantitative PCR at 24 h post infection (right panel). **b** cGAS-293FT cells infected with ΔNS1 influenza virus in the presence or absence of CBX (160 μM). IFN-β mRNA levels were assessed by quantitative PCR at 24 h post infection. **c** cGAS-293FT cells were transfected with the expression plasmid encoding HA-tagged IRF3. Twenty-four hours after transfection, the cells were infected with ΔNS1 influenza virus in the presence or absence of CBX (160 μM). Cell lysates were collected at 12 h post infection and blotted using the indicated antibodies. **d** Samples from cGAS-293FT cells transfected with siRNA targeting *connexin 43* (CX43) or control siRNA were blotted using the indicated antibodies (left panel) or intracellularly stained with CX43-specific antibody and analyzed by flow cytometry (right panel). **e** Schematic representation of experimental setup (left panel). cGAS-293FT cells transfected with siRNA targeting *connexin 43* (CX43) or control siRNA were infected with ΔNS1 influenza virus (purple). Six hours later, uninfected HEK293FT cells (red) were added to the ΔNS1 influenza virus-infected cGAS-293FT cells (purple) and co-cultured for additional 18 h. IFN-β mRNA levels were assessed by quantitative PCR at 24 h post infection (right panel). These data are from three independent experiments (**a**, **b**, **e**; mean ± s.e.m.). *P < 0.05, **P < 0.01, ***P < 0.001; (one-way ANOVA and Tukey’s test). Source data are provided as a Source Data file

Detection of cytosolic DNA by cGAS leads to the production of the second messenger cGAMP, which can pass through gap junctions to trigger STING-dependent antiviral immunity in bystander cells^12^. Thus, we next examined whether the innate immune signals are transferred from influenza virus-infected cells to neighboring cells. To this end, we infected cGAS-293FT cells with ΔNS1 influenza virus. At 9 h post infection, uninfected WT or STING-deficient HEK293FT cells were seeded and co-cultured with the virus-infected cGAS-293FT cells for an additional 15 h (Fig. 8a). We found that IFN-β gene expression was significantly reduced when ΔNS1 influenza virus-infected cGAS-293FT were co-cultured with STING-deficient HEK293FT cells (Fig. 8a). In addition, carbenoxolone (CBX), an inhibitor of gap junction, significantly inhibited the ΔNS1 virus-induced IFN-β gene expression and IRF3 phosphorylation without affecting the expression levels of M2 protein (Fig. 8b, c). Thus, we next examined the role of a gap junction protein connexin 43 (CX43) in the induction of IFN-β gene expression in response to influenza virus infection. Knockdown of CX43 in ΔNS1 influenza virus-infected cGAS-293FT cells significantly reduced IFN-β gene expression when ΔNS1 influenza virus-infected cGAS-293FT were co-cultured with CX43-sufficient HEK293FT cells (Fig. 8d, e). These results indicated that intercellular communication via gap junction is required to amplify STING-dependent antiviral immunity from influenza virus-infected cells to bystander cells.

### Effect of cGAS or STING deficiency on influenza virus replication

**Fig. 9:**
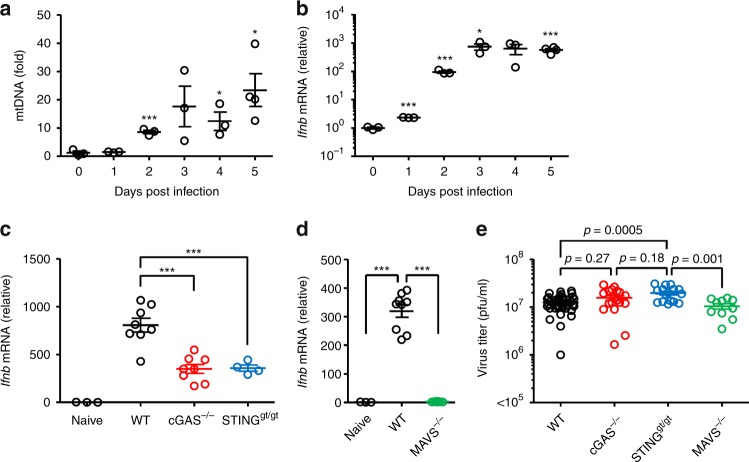
Effect of cGAS or STING deficiency on influenza virus replication in vivo. **a**, **b** WT mice were intranasally infected with 1,000 pfu of PR8 virus. The BAL fluids (**a**) and lung tissues (**b**) were collected at indicated time points. DNA was extracted from BAL fluids of mock- or influenza virus-infected mice using QIAquick Nucleotide Removal kit (QIAGEN). Cytosolic mtDNA was assessed by quantitative PCR (**a**). Total RNAs were extracted from the lung tissue of mock- or influenza virus-infected mice. IFN-β mRNA levels were assessed by quantitative PCR with GAPDH as an internal control (**b**). **c**, **d** WT, *cGAS*^−/−^, *Sting*^*gt/gt*^, and *MAVS*^−/−^ mice were intranasally infected with 1000 pfu of PR8 virus. Lung tissues were collected at 4 d post infection. Total RNAs were extracted from the lung tissue and IFN-β mRNA levels were assessed by quantitative PCR with GAPDH as an internal control. **e** WT (*n* = 37), *cGAS*^−/−^ (*n* = 18), *Sting*^*gt/gt*^ (*n* = 16), and *MAVS*^−/−^ (*n* = 10) mice were intranasally infected with 1000 pfu of PR8 virus. The BAL fluids were collected at 5 d post infection and viral titers were determined by standard plaque assay. These data are from three independent experiments (**a**–**d**; mean ± s.e.m.) or pooled from four independent experiments (**e**; mean ± s.e.m.). *P < 0.05, ***P < 0.001; (one-way ANOVA and Tukey’s test). Source data are provided as a Source Data file

Finally, we wished to determine the consequence of mtDNA release in the induction of cGAS/STING-dependent IFN-β gene expression in the lung tissue after influenza virus infection in vivo. In WT mice infected with influenza virus, cytosolic mtDNA release in the bronchoalveolar (BAL) fluid became apparent starting around day 2 p.i. and peaking around day 4 p.i. (Fig. 9a). Similarly, the levels of IFN-β mRNA in the lung tissue of WT mice became apparent starting around day 2 p.i. and peaking around day 4 p.i. (Fig. 9b). In addition, we found that the levels of IFN-β mRNA in the lung tissue of cGAS, STING, and MAVS KO mice infected with influenza virus were significantly reduced compared to WT mice (Fig. 9c, d). Thus, we next evaluated the impact of cGAS and STING deficiency on influenza virus replication in vivo. Consistent with a previous report^50^, the virus titers in the lung of infected mice were comparable between WT and MAVS KO mice at 5 d p.i. (Fig. 9e). Similarly, cGAS deficiency did not significantly affected the viral titer in the lung compared to WT mice (Fig. 9e). In contrast, the viral titer was significantly elevated in the lung of STING KO mice compared to WT mice (Fig. 9e). Although these data collectively indicate the importance of the STING-dependent signals in the induction of IFN-β gene expression and limiting influenza viral replication in vivo, it remains possible that cGAS and other DNA sensors induce redundant signaling pathways required for limiting viral replication in a cell type-specific manner.

## Discussion

The innate immune system utilizes pattern recognition receptors to detect pathogen-associated molecular patterns, such as viral nucleic acids. While TLR7 and RIG-I detect influenza viral RNA, the NLRP3 senses intracellular ionic fluxes following influenza virus infection. We previously demonstrated that the ion channel activity of viroporins, such as influenza virus M2 or EMCV 2B protein is essential for NLRP3 inflammasome activation^32,37^. Our findings here have identified a previously unknown mechanism by which influenza virus and EMCV stimulate mtDNA release into the cytosol through their viroporin activity. The cytosolic translocation of mtDNA in response to influenza virus or EMCV infection stimulates cGAS- and DDX41-dependent innate antiviral immune responses. Given that the viroporin-induced disturbance in the intracellular ionic milieu is accompanied by Mn^2+^ efflux from membrane-enclosed organelles, the ion channel activity of viroporins may be required for increasing the sensitivity of cGAS to dsDNA^51^.

**Fig. 10:**
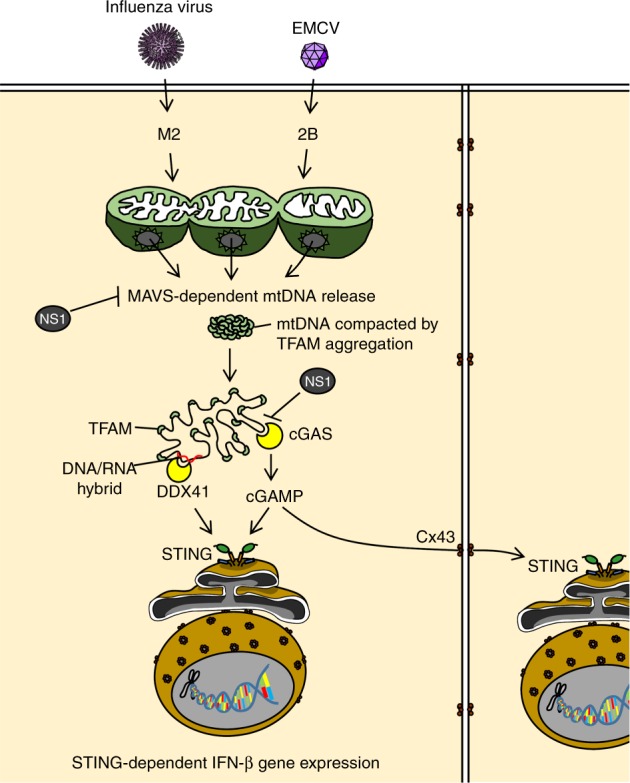
Proposed mechanism by which influenza virus regulates STING-dependent antiviral immunity. Ion channel activity of influenza virus M2 or EMCV 2B protein stimulates cytosolic mtDNA release in a MAVS-dependent manner. These cytosolic mtDNA could be packaged into distinct levels of higher-order structures depending on the ratio of TFAM to mtDNA. Cytosolic DNA sensors cGAS and DDX41 may recognize influenza virus-induced cytosolic mtDNA to stimulate STING-dependent IFN-β gene expression. The STING-dependent antiviral signaling was amplified in neighboring cells though gap junctions. The NS1 protein of influenza virus could associate with mtDNA to evade host DNA sensing pathways

Our data have demonstrated that the infection with ΔNS1 influenza virus enhances cytosolic mtDNA release and the STING-dependent IFN-β gene expression compared with that of WT virus. Several possible mechanisms could explain how the NS1 protein of influenza virus might inhibit mtDNA release into the cytosol and STING-dependent recognition of influenza virus infection. First, the NS1 protein of influenza virus might inhibit cytosolic translocation of mtDNA by inhibiting RIG-I/MAVS-dependent signals (Fig. 10). Indeed, we found that influenza virus stimulated cytosolic mtDNA release in a MAVS-dependent manner. In the case of SeV infection, the virus activated-IRF3 associates with Bax to translocate to the mitochondria and cause cytochrome c release^29^. In addition, previous studies have demonstrated that Bax/Bak play a critical role for mtDNA release into the cytosol^27,28^. Similarly, we found knockdown of Bax significantly reduced the cytosolic mtDNA release after influenza virus infection. Formation of Bak/Bax macropores elicits inner mitochondrial membrane herniation and stimulate mtDNA release into the cytosol^52^. These cytosolic mtDNA could be packaged into distinct levels of higher-order structures depending on the ratio of TFAM to mtDNA^53^ (Fig. 10). Given that cGAS preferentially binds incomplete nucleoid-like structures or U-turn DNA^54^, cytosolic U-turn DNA bridged by cross-strand binding of TFAM^53^ could play a major role in the induction of cGAS/STING-dependent IFN-β gene expression in response to influenza virus infection. Second, because the NS1 protein of influenza virus associated with mtDNA and inhibited detectable levels of cytosolic mtDNA, the NS1 protein may mask mtDNA from recognition by cytosolic DNA sensors (Fig. 10). Indeed, we found that treatment of pure cytosolic extracts of influenza virus-infected cells with proteinase K enhanced detectable levels of cytosolic mtDNA after influenza virus infection. Consequently, transfection of cGAS-293FT cells with proteinase K-treated cytosolic extracts from influenza virus-infected cells significantly enhanced IFN-β gene expression compared with untreated control extracts of influenza virus-infected cells, suggesting that the NS1 protein of influenza virus may associate with mtDNA to evade recognition by cytosolic DNA sensors (Fig. 10).

Influenza virus-induces type I IFNs (IFN-α/β) production in a STING-dependent but cGAS-independent manner through a membrane fusion process in human monocyte/macrophage-like cell line THP1^15^. In addition, knockdown of DDX41 in D2SC cells, a mouse myeloid DC line, has no effect on influenza virus-induced IFN-α/β production^41^. Furthermore, knockdown of STING in MEFs has no effect on influenza virus-induced IFN-β gene expression^16^. In contrast, our data have demonstrated that influenza virus stimulates cGAS-, DDX41-, and STING-dependent IFN-β gene expression in both mouse (primary lung fibroblasts) and human (HEK293FT and A549) cells. In addition, we found that influenza virus-induced high levels of cGAMP in STING-A549 cells or primary lung fibroblasts within 24 h post infection. Further, treatment of cells with CBX or knockdown of CX43 inhibited the STING-dependent IFN-β gene expression. These data collectively indicate that influenza virus infection stimulates STING-dependent pathways in a cell type-specific manner and that intercellular communication via gap junction plays an important role in spreading STING-dependent antiviral signals to bystander cells (Fig. 10).

Although cGAS was required to maximize IFN-β gene expression in the lung after influenza virus infection, cGAS deficiency did not significantly affected the viral titer in the lung compared to WT mice. In contrast, the STING-dependent signals were essential for limiting influenza virus replication in vivo. One possible explanation for this result is that cGAS and other DNA sensors induce redundant signaling pathways required for limiting influenza virus replication in the lung tissue. Another possibility is that STING-dependent translation inhibition could restrict influenza virus replication in vivo, independent of MAVS^16^. Since cGAS restricts viral replication of flaviviruses including dengue virus and West Nile virus^17,55^, the antiviral effects of the cGAS could be different for each RNA viruses^55^.

In summary, our finding substantially expand our understanding of how influenza virus and EMCV trigger mtDNA release into the cytosol and stimulate the cGAS- and DDX41-dependent innate antiviral immune responses. Because mitochondrial dsRNA released into the cytosol triggers MDA5-dependent innate antiviral signaling^56^, our results suggest a possible effect of viroporin-induced mitochondrial dysfunction in the induction of the MAVS-dependent innate antiviral immune responses. Better understanding of crosstalk between RNA and DNA sensing pathways in response to viral infection will aid the development of novel therapeutic strategies to treat viral infections and associated diseases.

## Methods

### Mice

Age- and sex-matched C57BL/6J obtained from Japan SLC, Inc. were used as WT controls. *cGAS*^−/−^, *Sting*^*gt/gt*^ (C57BL/6J-Tmem173gt/J), and *MAVS*^−/−^ mice were purchased from Jackson Laboratory (026554, 017537, and 008634 respectively). All animal experiments were performed in accordance with the regulations of the University of Tokyo Committee for Animal Care and Use and were approved by the Animal Experiment Committee of the Institute of Medical Science, the University of Tokyo.

### Cells and viruses

To prepare BMMs, bone marrows from the tibia and femur were obtained by flushing with Dulbecco’s modified Eagle’s medium (DMEM; Nacalai tesque). Bone marrow cells were cultured with DMEM supplemented with 10% heat-inactivated fetal bovine serum (FBS), L-glutamine and 30% L929 (provided by Dr. Akiko Iwasaki) supernatant containing the macrophage colony-stimulating factor at 37  °C for 5 days. For BMDCs, bone marrow from the tibia and femur was obtained as described above, and bone marrow cells were cultured in RPMI 1640 medium (Nacalai Tesque) containing 10% heat-inactivated FBS, L-glutamine and 5% J558L hybridoma cell (provided by Dr. Heung Kyu Lee) culture supernatant containing the granulocyte-macrophage colony-stimulating factor (GM-CSF) in 24-well plate for 5 days. The culture medium containing GM-CSF was replaced every other day. To obtain primary lung fibroblasts, lungs were minced using razor blades, and incubated in DMEM containing 0.25% trypsin and 0.5 mM EDTA at 37 °C for 30 min. Then, individual lungs were mechanically digested into single-cell suspensions by forcing them through a 70 μm cell strainer (BD) using a sterile syringe plunger. After centrifugation (200 × *g*, 5 min), pellets were resuspended in complete DMEM medium and cultured in 10-cm dish for 10 days. cGAS^−/−^ and STING^gt/gt^ MEFs were generated from embryos according to standard protocol. HEK293FT cells (#R70007, Invitrogen) and A549 cells (#CCL-185, ATCC) were maintained in DMEM supplemented with 10% FBS. Madin-Darby canine kidney (MDCK) cells (provided by Dr. Hideki Hasegawa) were grown in Eagle’s minimal essential medium (E-MEM; Nacalai Tesque) supplemented with 10% FBS.

WT A/PR8 influenza virus was grown in allantoic cavities of 10-d-old fertile chicken egg for 2d at 35 °C. Recombinant influenza viruses including WT A/PR8 influenza virus (termed rgPR8) used in this study were generated by using plasmid-based reverse genetics (Supplementary Table 2)^57^. Briefly, HEK293FT cells in collagen-coated 6-well plates were transfected with eight viral RNA-expressing plasmids together with plasmids expressing PA, PB1, PB2, and NP using transIT-293 (Takara Bio). At 48 h post-transfection, the cells were incubated for 15 min at 37 °C with 1 μg/ml acetylated trypsin. After centrifugation (600 × *g*, 5 min), cell supernatants were inoculated into MDCK cells in 6-well plates and cultured for 2–3 days in Opti-MEM containing 1 μg/ml acetylated trypsin to rescue the recombinant influenza virus. These recombinant influenza viruses were propagated in MDCK cells or MDCK cells stably expressing the influenza virus NS1 protein for 2 days at 37 °C^58^. EMCV used for all experiments was grown in L929 cells for 15 h at 37 ˚C. Human adenovirus type 5 was purchased from ATCC (VR-1516). Viruses were stored at −80 °C, and the viral titer was quantified in a standard plaque assay using MDCK cells for influenza virus and L929 cells for EMCV. The adenovirus titer was determined by 50% tissue culture infectious dose (TCID_50_) assay. Experimental infections were carried out in the BSL-2 facility at the Institute of Medical Science, the University of Tokyo. The ethics committees of the Institute of Medical Science, the University of Tokyo approved all of the experimental protocols.

### Influenza virus infection

Cells were infected with influenza virus at a MOI of 1–10 for 1 h at 37 °C, and cultured with complete DMEM for 24 h without trypsin.

For intranasal infection, mice were fully anesthetized by intraperitoneal injection of pentobarbital sodium (Somnopentyl, Kyoritsu Seiyaku Co., Ltd., Tokyo, Japan) and then infected by intranasal application of 30 μl of virus suspension (1000 pfu in PBS).

### Antibodies

The anti-EMCV 2B antibody (1:1000) was generated by immunizing a rabbit with the C-terminal region of 2B protein, which was supplied by peptide synthesis (FITPPPRFPTISL). Monoclonal antibody against influenza A virus M2 protein (14C2, Cat#ab5416; 1:1000) and anti-dsDNA (35I9 DNA, Cat#ab27156; 1:600) were purchased from abcam. Monoclonal antibody against Flag (M2, Cat#F1804; 1:1000), rabbit polyclonal antibodies against Flag (Cat#F7425; 1:10,000) and calnexin (Cat#C4731; 1:2000) were obtained from Sigma-Aldrich. Anti-GFP (GF200, Cat#04363-66; 1:10,000) was from Nacalai Tesque (Kyoto, Japan). Anti-cGAS (D1D3G, Cat#15102; 1:1000), anti-DDX41 (D3F1Z, Cat#15076; 1:1000), anti-STING (D2P2F, Cat#13647; 1:1000), anti-MAVS (Cat#3993; 1:1000), and anti-TFAM (D5C8, Cat#8076; 1:1000) were purchased from Cell Signaling Technology (Danvers, MA, USA). Anti-influenza virus NS1 (NS1-23-1, Cat#sc-130568; 1:1000), anti-HA (F-7, Cat#sc-7392; 1:1000), anti-myc (9E10, Cat#sc-40; 1:1000), anti-tubulin (DM1A, Cat#sc-32293; 1:2000), anti-Tom20 (FL-145, Cat#sc-11415; 1:1000), anti-Mfn2 (XX-1, Cat#sc-100560; 1:1000), and anti-connexin 43 (F-7, Cat#sc-271837; 1:1000) were purchased from Santa Cruz Biotechnology. Mouse monoclonal antibody against RIG-I (Alme-1, Cat#AG-20B-0009-C100; 1:1000) was from AdipoGen.

### Lentiviral vectors

To generate lentiviruses expressing the cGAS protein, the full-length cDNA encoding the cGAS protein was cloned into the pLenti6.3/V5-TOPO vector (Invitrogen). HEK293FT cells cultured in a collagen-coated 10-cm dish were transfected with 3 μg of cGAS-expressing pLenti6.3/V5-TOPO vector together with ViraPower Packaging Mix (Invitrogen) using Lipofectamine 2000 (Invitrogen). The culture medium was replaced with fresh medium 24 h later. At 72–96 h posttransfection, the lentivirus-containing supernatants were harvested. A lentivirus encoding an irrelevant protein (EGFP) served as a control^58^. The stock virus, containing Polybrene (10 μg/ml), was then inoculated into HEK293FT cells. The culture medium was replaced with fresh medium 24 h later. Finally, the cells were cultured for 2–3 weeks in complete medium containing blasticidin (10 μg/ml) to kill nontransduced cells.

### Establishment of gene-knockout cell lines

Each of the target sequence of single-guide RNA was cloned into pX458 (addgene, #48138), followed by annealing of oligonucleotide pairs (Supplementary Table 3). HEK293FT or A549 cells were transfected with resulting plasmid and incubated for 2~3 days. EGFP positive cells were sorted by FACS and formed a single colony. Gene deficiency was confirmed by sequencing and immunoblotting. A representative single clone for each gene was used for experiments.

### Establishment of mtDNA-depleted ρ0 cell lines

HEK293FT or cGAS-293FT cells were cultured in DMEM supplemented with 10% FBS, pyruvate (100 μg/mL), ethidium bromide (EtBr) (500 ng/mL), and uridine (50 μg/mL) for 2 wks, as previously described^59^. The depletion of mtDNA was confirmed by quantitative PCR using the following primers: human mtDNA forward, 5′-cctagggataacagcgcaat-3′, and reverse, 5′-tagaagagcgatggtgagag-3′; human β-actin forward, 5′-ctggaacggtgaaggtgaca-3′, and reverse, 5′-aagggacttcctgtaacaatgca-3′ (Supplementary Table 4)^60,61^. The relative mtDNA amounts were shown as a ratio of mtDNA to nuclear DNA encoding β-actin

### Detection of mtDNA in cytosolic extracts

BAL fluid was collected by washing the trachea and lungs twice by injecting a total of 2 ml buffer containing 150 mM NaCl, 50 mM HEPES pH 7.4, and 20 μg/ml digitonin (Nacalai Tesque) for measurement of cytosolic mtDNA release in the lung. HEK293FT, A549, primary lung fibroblasts or MEFs were resuspended in 500 μl buffer containing 150 mM NaCl, 50 mM HEPES pH 7.4, and 20 μg/ml digitonin (Nacalai Tesque). The homogenates were incubated on an end-over-end rotator for 10 min. After centrifugation (1000 × *g*, 3 min) three times, the supernatants were transferred to fresh tubes and centrifuged at 17,000 × *g* for 10 min. Cytosolic mtDNA was isolated from these pure cytosolic fractions using QIAquick Nucleotide Removal kit (QIAGEN). Total mtDNA was isolated from whole-cell extracts using QIAamp DNA Mini Kit (QIAGEN). Quantitative PCR was performed on both pure cytosolic fractions and whole-cell extracts using mtDNA primers: human mtDNA forward, 5′-cctagggataacagcgcaat-3′, and reverse, 5′-tagaagagcgatggtgagag-3′; mouse mtDNA forward, 5′- gccccagatatagcattccc-3′, and reverse, 5′-gttcatcctgttcctgctcc-3′ (Supplementary Table 4)^60,62^. The relative cytosolic mtDNA levels were normalized to total mtDNA amounts. The value in mock-infected or EGFP-transfected cells was set to 1.

### Quantitative PCR

Total RNA was extracted from cells using TRIzol reagent (Invitrogen) and reverse transcribed into cDNA using SuperScript III reverse transcriptase (Invitrogen) with an oligo (dT) primer. SYBR Premix Ex Taq II (TaKaRa) and a LightCycler instrument (Roche Diagnostics) were used for quantitative PCR with the following primers: human IFN-β forward, 5′-ctcctggctaatgtctatca-3′, and reverse, 5′-gcagaatcctcccataatat-3′; human β-actin forward, 5′-ctggaacggtgaaggtgaca-3′, and reverse, 5′- aagggacttcctgtaacaatgca-3′; mouse IFN-β forward, 5′-gcactgggtggaatgagactattg-3′, and reverse, 5′-ttctgaggcatcaactgacaggtc-3′; mouse GAPDH forward, 5′- accacagtccatgccatca-3′, and reverse, 5′-tccaccaccctgttgctgta-3′^37,61,62^; influenza virus NP forward, 5′-agaacatctgacatgaggac-3′, and reverse, 5′-gtcaaaggaaggcacgatc-3′ (Supplementary Table 4).

### IFN-β reporter assay

cGAS-293FT cells seeded on 24-well cluster plates were cotransfected with 100 ng of p125-luc (a gift from T. Taniguchi, University of Tokyo), 2.5 ng of phRL-TK, and 100 ng of poly(dA:dT) or mtDNA together with 100 ng of pCA7-EGFP, pCA7-Flag-NS1, or pCA7-Flag-NS1 (R38A/K41A)^58,61^. After 24 h of transfection, cells were lysed in passive lysis buffer (Promega) and examined for luciferase activity using the dual-luciferase reporter assay system (Promega). Data were normalized for transfection efficiency against Renilla luciferase activity.

### ELISA

Cell-free supernatants were collected at 24 h p.i. The supernatants were analyzed for the presence of IL-1β using an enzyme-linked immunosorbent assay (ELISA) utilizing paired antibodies (eBioscience)^32^. cGAMP ELISA (Cayman Chemical) was performed according to the manufacturer’s instructions.

### Flow cytometry

Cells were fixed and permeabilized using a Cytofix/Cytoperm kit (BD Biosciences), and intracellularly stained with mouse monoclonal anti-dsDNA (35I9 DNA, Cat#ab27156; Abcam; 1:600), anti-connexin 43 (F-7, Cat#sc-271837; Santa Cruz; 1:100) antibodies, or rabbit polyclonal antibodies against influenza virus M2 (1:600) or EMCV 2B (1:600) proteins followed by FITC-labeled goat anti-mouse IgG (Cat#405305; BioLegend; 1:300), PE-labeled goat anti-mouse IgG (Cat#405307; BioLegend; 1:300), or FITC-labeled donkey anti-rabbit IgG (Cat#406403; BioLegend; 1:300). Flow cytometric analysis was performed with a FACSVerse flow cytometer (BD Biosciences). The final analysis and graphical output were performed using FlowJo software (Tree Star, Inc.). All samples were gated based on forward scatter (FSC) and side scatter (SSC) to gate out cellular debris or dead cells.

### Confocal microscopy

STING-A549 cells were seeded onto coverslips in 24-well cluster plates and infected with PR8 virus or EMCV. At indicated time points, cells were fixed and permeabilized with PBS containing 4% formaldehyde and 1% Triton X-100. Cells were then washed with PBS and incubated with anti-influenza virus NS1 (Cat#GTX125990; GeneTex; 1:1000), anti-dsDNA (AC-30-10, Cat#CBL186; Chemicon International, Inc.; 1:500), or anti-Tom20 (FL-145, Cat#sc-11415; Santa Cruz; 1:200) antibodies, followed by incubation with Alexa Fluor 488-conjugated donkey anti-mouse IgG (H+L) (Cat#A21202; Life Technologies; 1:5000), Alexa Fluor 488-conjugated goat anti-mouse IgM (Cat#ab150121; Abcam; 1:5000), or Alexa Fluor 568-conjugated goat anti-rabbit IgG (H+L) antibodies (Cat#A11036; Life Technologies; 1:5000). Stained cells were observed under a confocal microscope (LSM5; Zeiss).

### Coimmunoprecipitation (co-IP) and western blot analysis

For co-IP of mtDNA-protein complexes, subconfluent monolayers of HEK293FT cells in collagen-coated 10-cm dishes were transfected with 12 μg of pCA7-EGFP, pCA7-Flag-M2, pCA7-Flag-NS1, or pCA7-Flag-NS1_38/41A_^58^. At 24 h posttransfection, cells were infected with ΔNS1 influenza virus for 24 h. Pure cytosolic extracts prepared from digitonin extracts were incubated for 60 min at 4 °C with protein G-Sepharose (GE Healthcare AB), which had been pretreated with an anti-Flag (M2, Cat#F1804; Sigma) or normal mouse IgG1 (Cat#sc-3877; Santa Cruz) antibody overnight at 4 °C. Complexes were obtained by centrifugation and washed five times with coimmunoprecipitation buffer (50 mM Tris [pH 7.5], 150 mM NaCl, 1% Triton X-100, 1 mM EDTA). mtDNA was isolated from these protein complexes using QIAquick Nucleotide Removal kit (QIAGEN). Quantitative PCR was performed using mtDNA primers (Supplementary Table 4).

For co-IP of ISD-protein complexes, subconfluent monolayers of cGAS-293FT cells in collagen-coated 10-cm dishes were transfected with 12 μg of biotin-labeled ISD. At 9 h posttransfection, cells were infected with WT A/PR8 influenza virus. At 15 h postinfection, the cells were washed with PBS and lysed in 1 ml PBS by 30 repetitive pipetting through 1 ml syringes and 21-gauge needles. The homogenate was centrifuged at 800 × *g* for 10 min at 4 °C. The supernatant was incubated for 60 min at 4 °C with streptavidin agarose (Thermo Scientific). Complexes were obtained by centrifugation and washed five times with PBS. The polypeptides within the precipitated complexes were fractionated by SDS-polyacrylamide gel electrophoresis (PAGE) (10–15% gels) and electroblotted onto polyvinylidene difluoride (PVDF) membranes (Immobilon-P; Millipore). The membranes were incubated with mouse anti-influenza A virus NS1 (NS1–23–1, Cat#sc-130568; Santa Cruz; 1:1000), rabbit anti-cGAS (D1D3G, Cat#15102; Cell Signaling Technology; 1:1000), rabbit anti-DDX41 (D3F1Z, Cat#15076; Cell Signaling Technology; 1:1000), rabbit anti-TFAM (D5C8, Cat#8076; Cell Signaling Technology; 1:1000), or rabbit anti-Flag (Cat#F7425; Sigma; 1:10,000) antibody, followed by incubation with horseradish peroxidase-conjugated anti-mouse IgG (Cat#115-035-003; Jackson Immuno Research Laboratories; 1:10,000) or anti-rabbit IgG (Cat#G21234; Life Technologies; 1:10,000). The PVDF membranes were then treated with Chemi-Lumi One Super (Nacalai Tesque) to elicit chemiluminescent signals, which were detected and visualized using an LAS-4000 Mini apparatus (GE Healthcare). All full-length western blots are available in Source Data file.

### Statistical analysis

Statistical significance was tested using nonparametric one-way analysis of variance (ANOVA), using PRISM software (version 8; GraphPad software). *P*-values of < 0.05 were considered statistically significant.

## Supplementary information

Supplementary Information

Source Data

## Data Availability

The source data underlying Figs. 1a–c, f–j, 2, 3a, b, e–g, 4–9 and Supplementary Figs. 1b, c, 2, 3, 4b, c, e, 5–14, 17–20 are provided as a Source Data file. All other data are available from the corresponding author upon reasonable requests.
