# Supplementary material for: Influenza A virus M2 protein triggers mitochondrial DNA-mediated antiviral immune responses
Source: Nat Commun. 2019 Oct 11;10:4624. doi: 10.1038/s41467-019-12632-5 (PMC6789137; doi:10.1038/s41467-019-12632-5)
Supplement: Supplementary file 1 — Supplementary Information [file 41467_2019_12632_MOESM1_ESM.pdf]

Supplementary Information for

Influenza A virus M2 protein triggers mitochondrial DNA-mediated antiviral immune responses.

Moriyama et al.

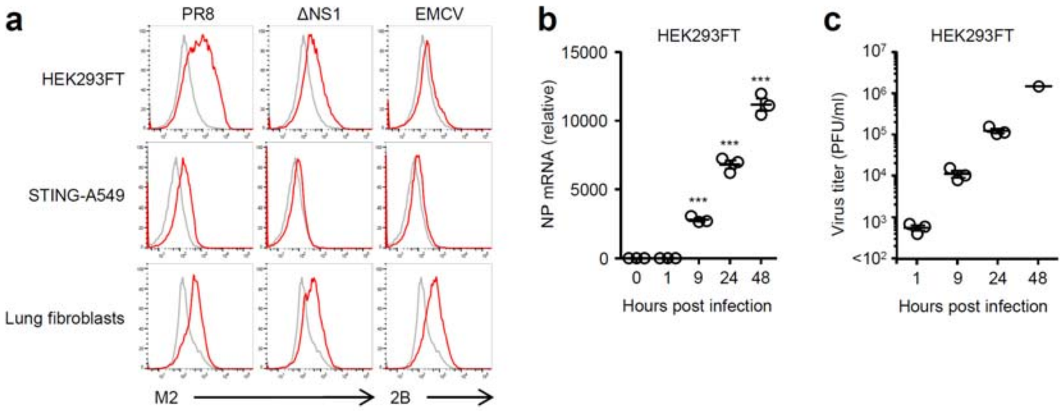

Supplementary Figure 1

Infectivity of Influenza virus and EMCV in HEK293FT, STING-A549 and mouse primary lung fibroblasts.

a, HEK293FT, STING-A549, and mouse primary lung fibroblasts were infected with PR8, ΔNS1, or EMCV without trypsin. Twenty-four hours after infection, the cells were fixed and permeabilized using a Cytofix/Cytoperm kit (BD Biosciences), and intracellulary stained with rabbit polyclonal antibodies against influenza virus M2 or EMCV 2B proteins followed by FITC donkey anti-rabbit IgG (BioLegend). **b,c,** HEK293FT cells were infected with WT A/PR8 influenza virus at an MOI of 0.01 without trypsin. Total RNA was extracted at indicated time points. Influenza virus nucleoprotein (NP) mRNA levels were assessed by quantitative PCR with β-actin as an internal control (**b**). Supernatants were collected at indicated time points and analyzed for viral titer by standard plaque assay using MDCK cells (**c**). These data are from two independent experiments (mean ± s.e.m.). ****P* < 0.001; (one-way ANOVA and Tukey's test). Source data are provided as a Source Data file.

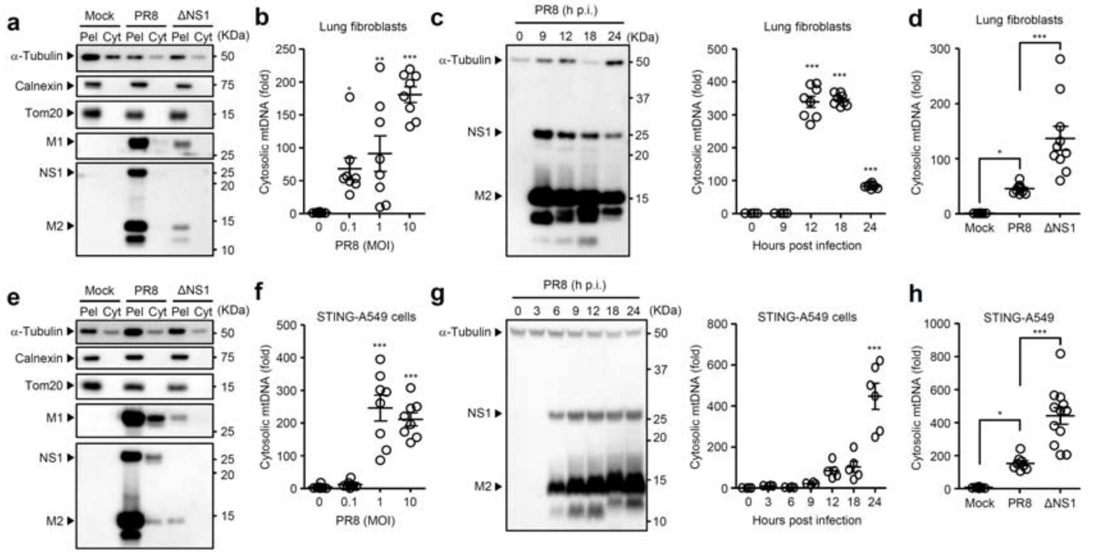

Supplementary Figure 2

Influenza virus stimulates cytosolic mtDNA release in lung fibroblasts and STING-A549 cells.

a, Influenza virus-infected lung fibroblasts were subjected to digitonin fractionation and pellets (Pel) or cytosolic extracts (Cyt) were analyzed by Western blotting using the indicated antibodies. **b,** Primary lung fibroblasts were infected with PR8 virus at indicated MOIs. DNA was extracted from digitonin extracts of mock- or influenza virus-infected cells. Cytosolic mtDNA was assessed by quantitative PCR. **c,** Primary lung fibroblasts were infected with PR8 virus at MOI of 10. Cell lysates were collected at indicated time points and analyzed by immunoblotting with indicated antibodies (left panel). DNA was extracted from digitonin extracts at indicated time points. Cytosolic mtDNA was assessed by quantitative PCR (right panel). **d**, Primary lung fibroblasts were infected with PR8 or ΔNS1 influenza virus at MOI of 10. DNA was extracted from digitonin extracts of mock- or influenza virus-infected cells. Cytosolic mtDNA was assessed by quantitative PCR. **e,** Influenza virus-infected STING-A549 were subjected to digitonin fractionation and pellets (Pel) or cytosolic extracts (Cyt) were analyzed by Western blotting using the indicated antibodies. **f,** STING-549 cells were infected with PR8 virus at indicated MOIs. DNA was extracted from digitonin extracts of mock- or influenza virus-infected cells. Cytosolic mtDNA was assessed by quantitative PCR. **g,** STING-A549 cells were infected with PR8 virus at MOI of 10. Cell lysates were collected at indicated time points and analyzed by immunoblotting with indicated antibodies (left panel). DNA was extracted from digitonin extracts at indicated time points. Cytosolic mtDNA was assessed by quantitative PCR (right panel). **h**, STING-A549 cells were infected with PR8 or ΔNS1 influenza virus at MOI of 10. DNA was extracted from digitonin extracts of mock- or influenza virus-infected cells. Cytosolic mtDNA was assessed by quantitative PCR. These data are from two independent experiments (mean ± s.e.m.). *P < 0.05, **P < 0.01, ****P* < 0.001; (one-way ANOVA and Tukey's test). Source data are provided as a Source Data file.

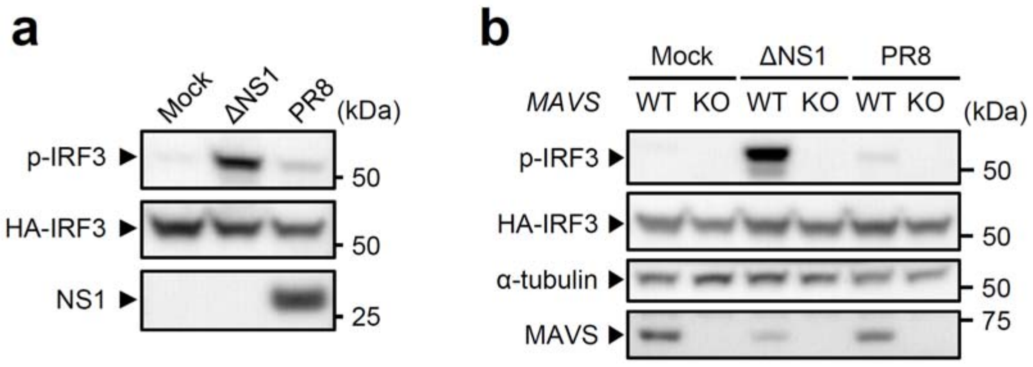

Supplementary Figure 3

Influenza virus induces phosphorylation of IRF3 in a MAVS-dependent manner.

a,b, WT or MAVS-deficient HEK293FT cells were transfected with the plasmid encoding HA-tagged IRF3. Twenty-four hours after transfection, the cells were infected with WT or ΔNS1 influenza virus. Cell lysates were collected at 12 h post infection and blotted using the indicated antibodies. Data was representative of three independent experiments.

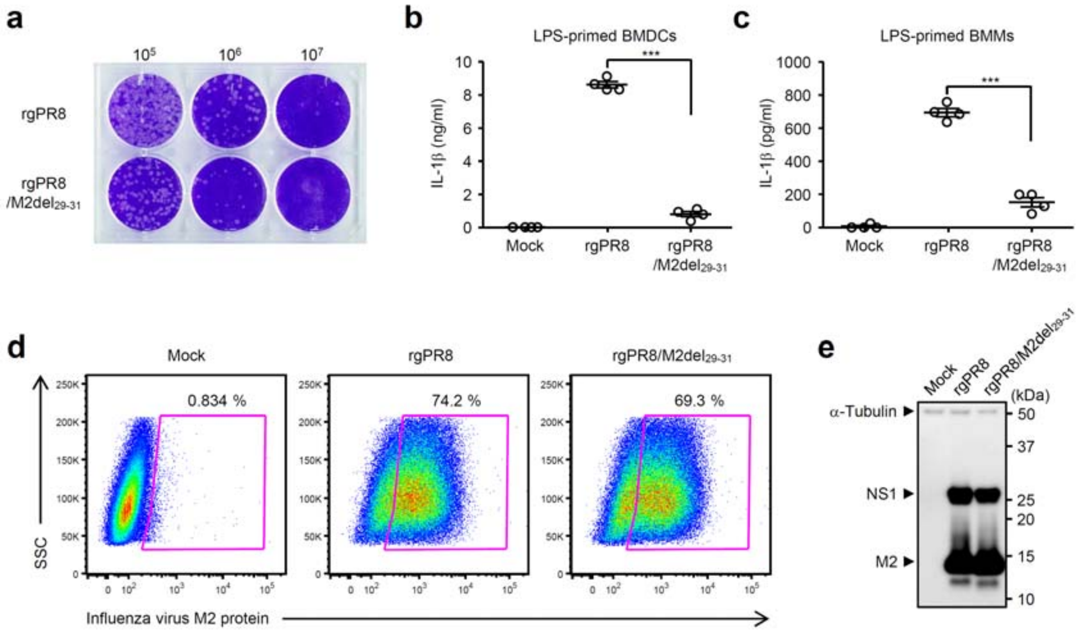

Supplementary Figure 4

Characterization of a rgPR8/M2del29-31 virus.

a, Virus titers of WT (rgPR8) or M2del29-31 virus (rgPR8/M2del29-31) were measured by plaque assay using MDCK cells in six-well plates with serial tenfold dilution of the stock virus. **b,c,** LPS-primed bone marrow-derived dendritic cells (BMDCs) (**b**) or macrophages (BMMs) (**c**) were infected with WT (rgPR8) or M2del29-31 virus (rgPR8/M2del29-31) for 24 h. Cell-free supernatants were collected and analyzed for IL-1β by ELISA. **d,e,** HEK293FT cells were infected with WT (rgPR8) or M2del29-31 virus (rgPR8/M2del29-31). Cell surface expression of M2 protein was assessed at 24 h post infection by flow cytometry using M2-specific antibody (14C2) (**d**). Cell lysates were collected at 24 h post infection and blotted using the indicated antibodies (**e**). These data are from two independent experiments (**b,c;** mean ± s.e.m.). **P < 0.01, ***P < 0.001; (one-way ANOVA and Tukey's test). Source data are provided as a Source Data file.

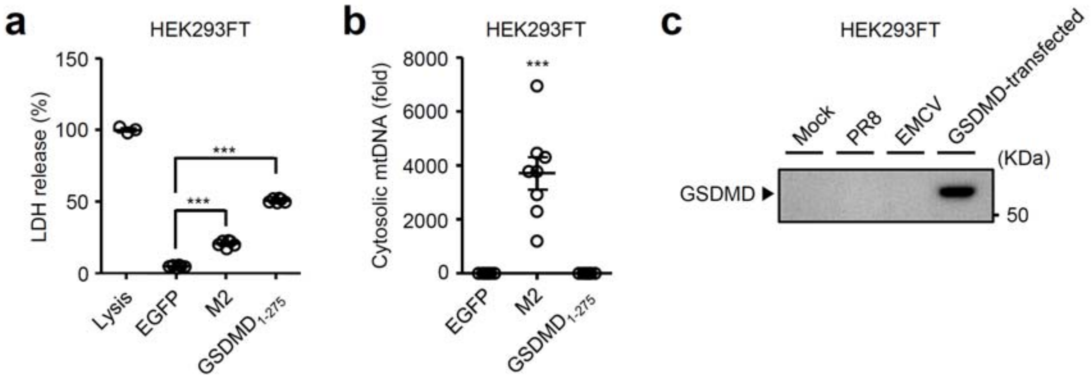

Supplementary Figure 5

GSDMD does not stimulate cytosolic mtDNA release.

a,b, HEK293FT cells were transfected with the expression plasmid encoding EGFP, influenza virus M2, or GSDMD residues 1-275 ($GSDMD_{1\text{-}275}$). LDH activity was measured at 24 h post transfection (**a**). Cytosolic mtDNA was assessed by quantitative PCR at 24 h post transfection (**b**). **c,** HEK293FT cells were infected with PR8 or EMCV. Cell lysates were collected at 24 h post transfection and blotted using an anti-GSDMD antibody (64-Y). Flag-tagged GSDMD-transfected cell lysates were used as a positive control. These data are from two independent experiments (mean ± s.e.m.). ***$P < 0.001$; (one-way ANOVA and Tukey's test). Source data are provided as a Source Data file.

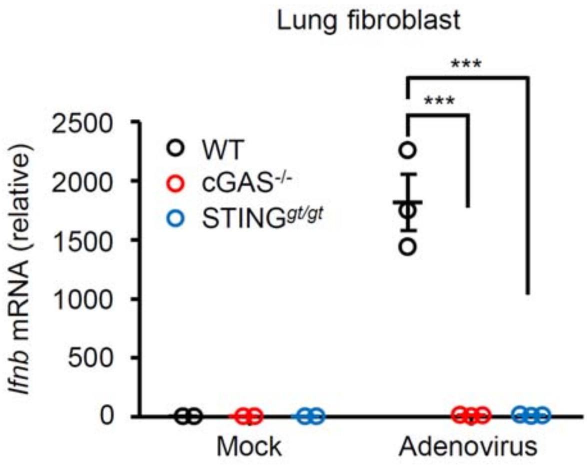

Supplementary Figure 6

Adenovirus stimulates cGAS- and STING-dependent IFN-β gene expression in mouse lung fibroblast.

Primary lung fibroblast prepared from WT, cGAS-, and STING-deficient mice were infected with adenovirus. IFN-β mRNA levels were assessed by quantitative PCR with GAPDH as an internal control. Source data are provided as a Source Data file.

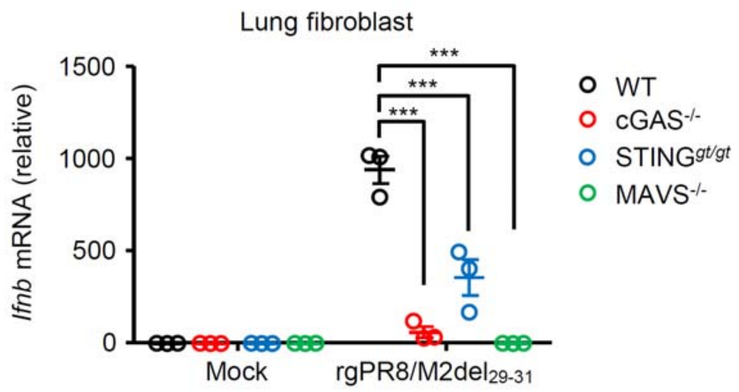

Supplementary Figure 7

rgPR8/M2del$_{29\text{-}31}$ virus stimulates cGAS- and STING-dependent IFN-β gene expression in mouse lung fibroblast.

Primary lung fibroblast prepared from WT, cGAS-, STING-, and MAVS-deficient mice were infected with rgPR8/M2del$_{29\text{-}31}$ virus. IFN-β mRNA levels were assessed by quantitative PCR with GAPDH as an internal control. Source data are provided as a Source Data file.

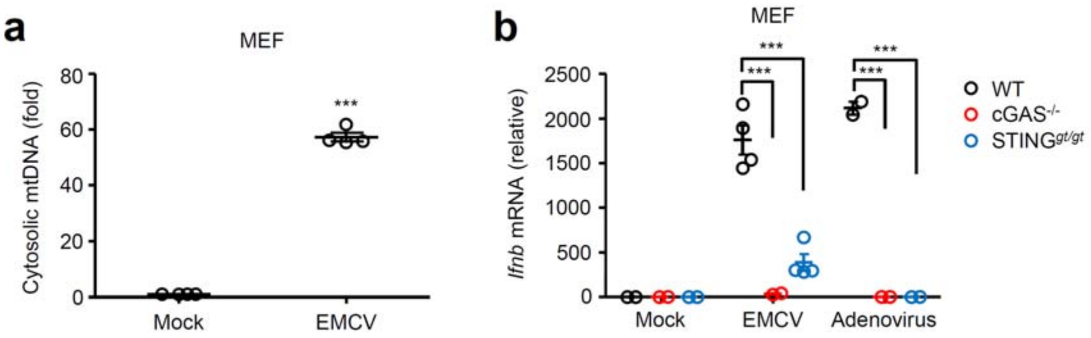

Supplementary Figure 8

EMCV stimulates cGAS/STING-dependent IFN-β mRNA expression in MEFs.

a, WT MEFs were infected with EMCV. Cytosolic mtDNA was assessed by quantitative PCR at 24 h post infection. **b,** MEFs prepared from WT, cGAS-, and STING-deficient mice were infected with adenovirus or EMCV for 24 h. IFN-β mRNA levels were assessed by quantitative PCR with GAPDH as an internal control. These data are from three independent experiments (mean ± s.e.m.). ***$P < 0.001$; (one-way ANOVA and Tukey's test). Source data are provided as a Source Data file.

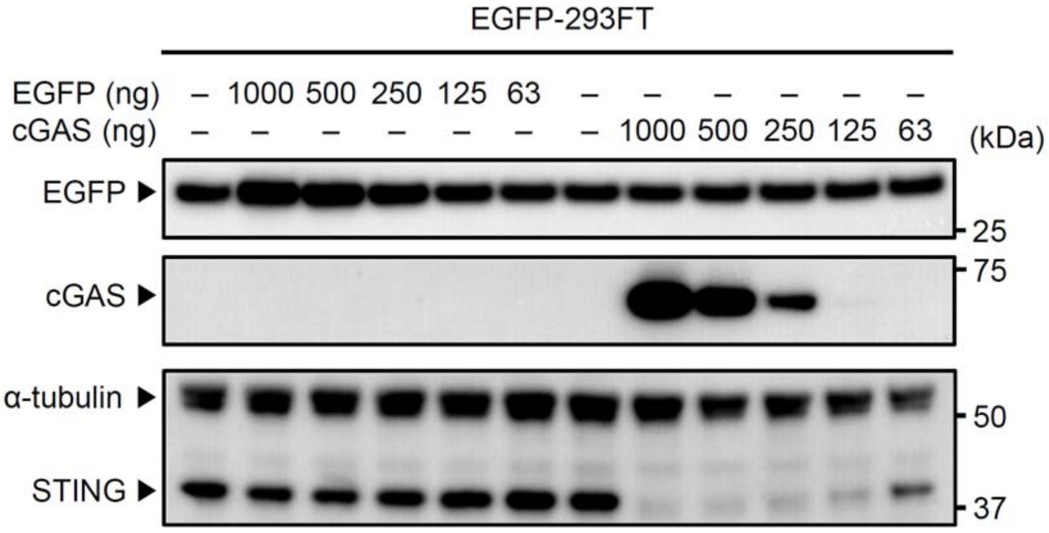

Supplementary Figure 9

Overexpression of cGAS reduced the expression levels of STING.

EGFP-293FT cells were transfected with indicated amounts of the expression plasmid encoding EGFP or HA-tagged cGAS. Cell lysates were collected at 24 h post transfection and blotted using anti-HA or indicated antibodies. Data was representative of three independent experiments.

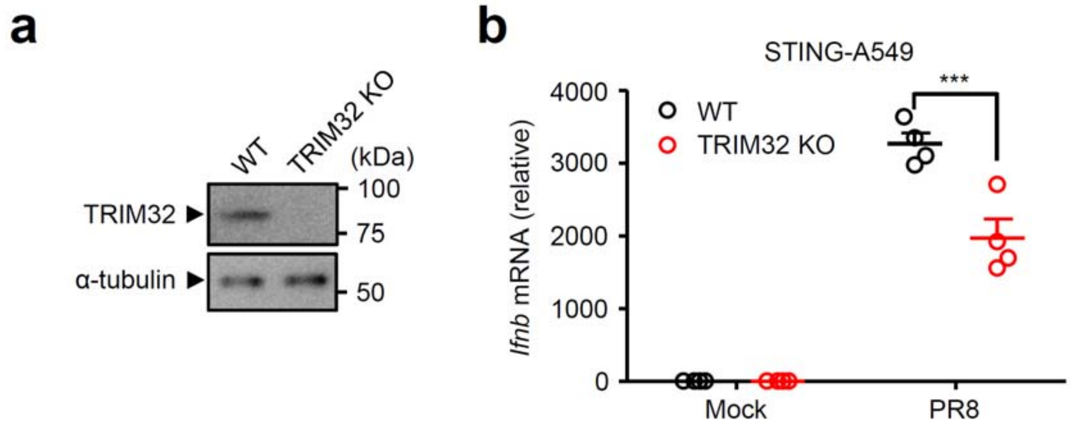

Supplementary Figure 10

TRIM32 is required for influenza virus-induced IFN-β gene expression.

a, Samples from WT or TRIM32 KO STING-A549 cells were blotted using the indicated antibodies. **b,** WT or TRIM32 KO STING-A549 cells were infected with influenza virus for 24 h. IFN-β mRNA levels were assessed by quantitative PCR with β-actin as an internal control. These data are from two independent experiments (**b;** mean ± s.e.m.). ***P < 0.001; (one-way ANOVA and Tukey's test). Source data are provided as a Source Data file.

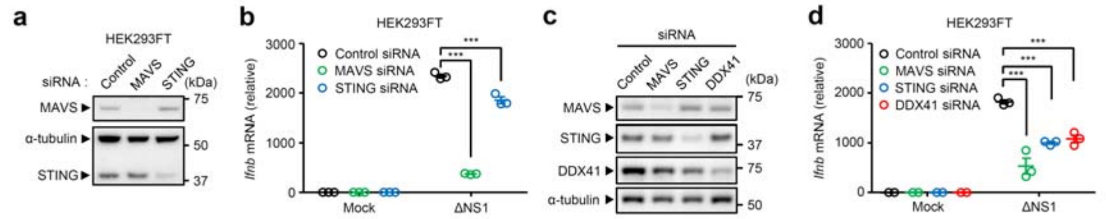

Supplementary Figure 11

ΔNS1 influenza virus stimulates DDX41-dependent IFN-β mRNA expression in HEK293FT cells.

a, Samples from HEK293FT cells transfected with siRNA targeting *MAVS*, *STING*, or control siRNA were blotted using the indicated antibodies. **b,** HEK293FT cells transfected with siRNA targeting *MAVS*, *STING*, or control siRNA were infected with ΔNS1 influenza virus for 24 h. IFN-β mRNA levels were assessed by quantitative PCR with β-actin as an internal control. **c**, Samples from HEK293FT cells transfected with siRNA targeting *MAVS*, *STING*, *DDX41*, or control siRNA were blotted using the indicated antibodies. **d,** HEK293FT cells transfected with siRNA targeting *MAVS*, *STING*, *DDX41*, or control siRNA were infected with ΔNS1 influenza virus for 24 h. IFN-β mRNA levels were assessed by quantitative PCR with β-actin as an internal control. These data are from three independent experiments (**b,d;** mean ± s.e.m.). ***$P < 0.001$; (one-way ANOVA and Tukey's test). Source data are provided as a Source Data file.

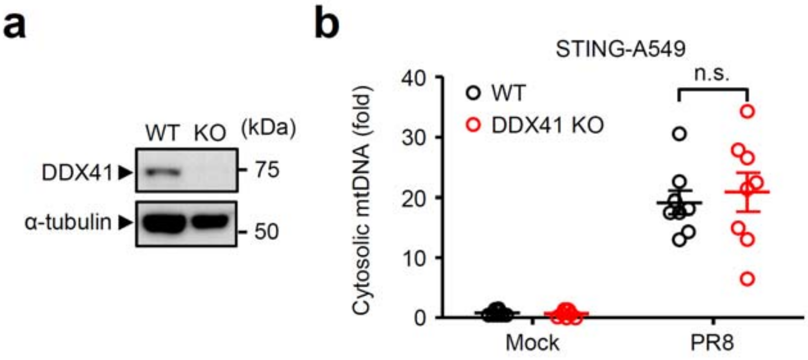

Supplementary Figure 12

mtDNA release is intact in DDX41 KO STING-A549 cells after influenza virus infection.

a, Samples from WT or DDX41 KO STING-A549 cells were blotted using the indicated antibodies. **b,** WT or DDX41 KO STING-A549 cells were infected with influenza virus for 24 h. Cytosolic mtDNA was assessed by quantitative PCR. These data are from two independent experiments (**b;** mean ± s.e.m.). n.s., not significant. Source data are provided as a Source Data file.

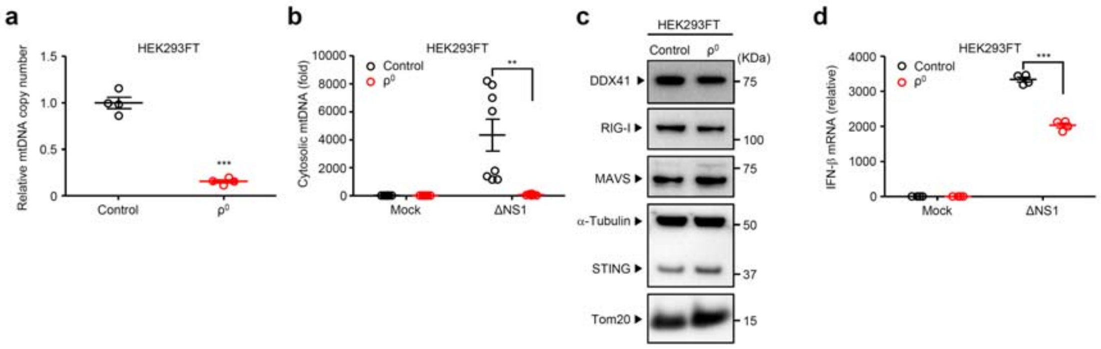

Supplementary Figure 13

ρ0 HEK293FT cells reduces IFN-β gene expression after ΔNS1 influenza virus infection.

a, Total mtDNA copy number was measured by quantitative PCR. **b,** Control or ρ0 HEK293FT cells were infected ΔNS1 influenza virus for 24 h. Cytosolic mtDNA was assessed by quantitative PCR. **c,** Samples from Control or ρ0 HEK293FT cells were blotted using the indicated antibodies. **d,** Control or ρ0 HEK293FT cells were infected ΔNS1 influenza virus for 24 h. IFN-β mRNA levels were assessed by quantitative PCR with β-actin as an internal control. These data are from three independent experiments (**a,b,d;** mean ± s.e.m.). $**P < 0.01$, $***P < 0.001$; (one-way ANOVA and Tukey's test). Source data are provided as a Source Data file.

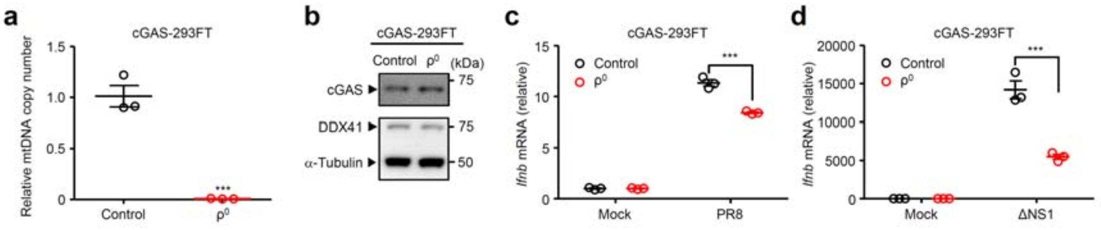

Supplementary Figure 14

ρ0 cGAS-293FT cells reduces IFN-β gene expression after WT or ΔNS1 influenza virus infection.

a, Total mtDNA copy number was measured by quantitative PCR. **b,** Samples from Control or ρ0 cGAS-293FT cells were blotted using the indicated antibodies. **c,d,** Control or ρ0 cGAS-293FT cells were infected with WT A/PR8 (**c**) or ΔNS1 influenza virus (**d**) for 24 h. IFN-β mRNA levels were assessed by quantitative PCR with β-actin as an internal control. These data are from three independent experiments (**a,c,d;** mean ± s.e.m.). ***P < 0.001; (one-way ANOVA and Tukey's test). Source data are provided as a Source Data file.

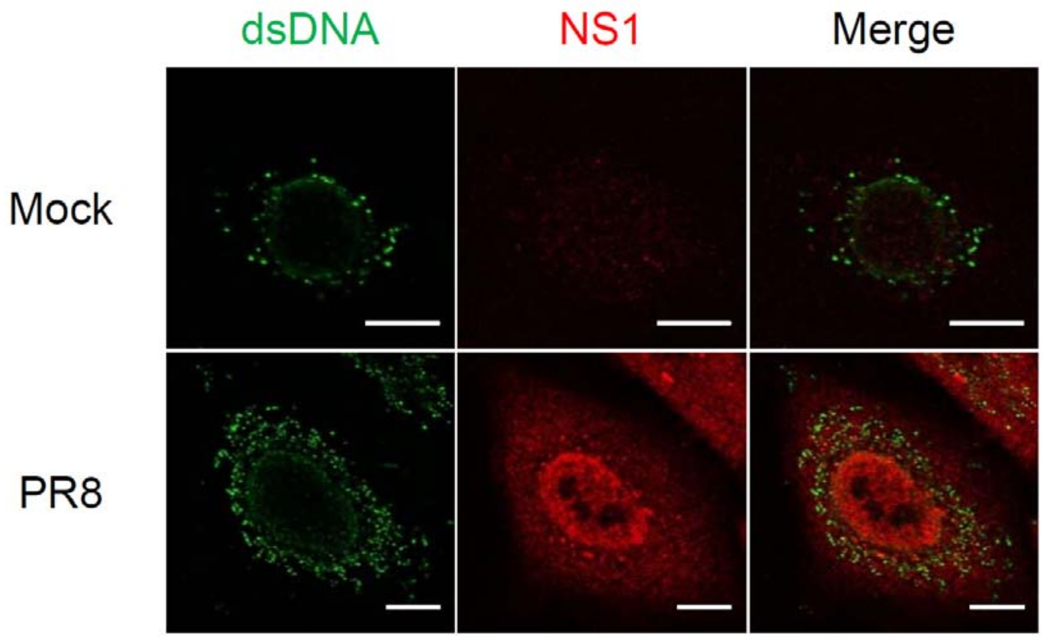

Supplementary Figure 15

The NS1 protein of influenza virus does not co-localize with dsDNA in cytosol of influenza virus-infected cells.

STING-A549 cells were infected with influenza virus. At 24 h post infection, cells were stained with anti-dsDNA (AC-30-10) and anti-NS1 (GTX125990) antibodies and analyzed by confocal microscopy. Scale bars, 10 μm.

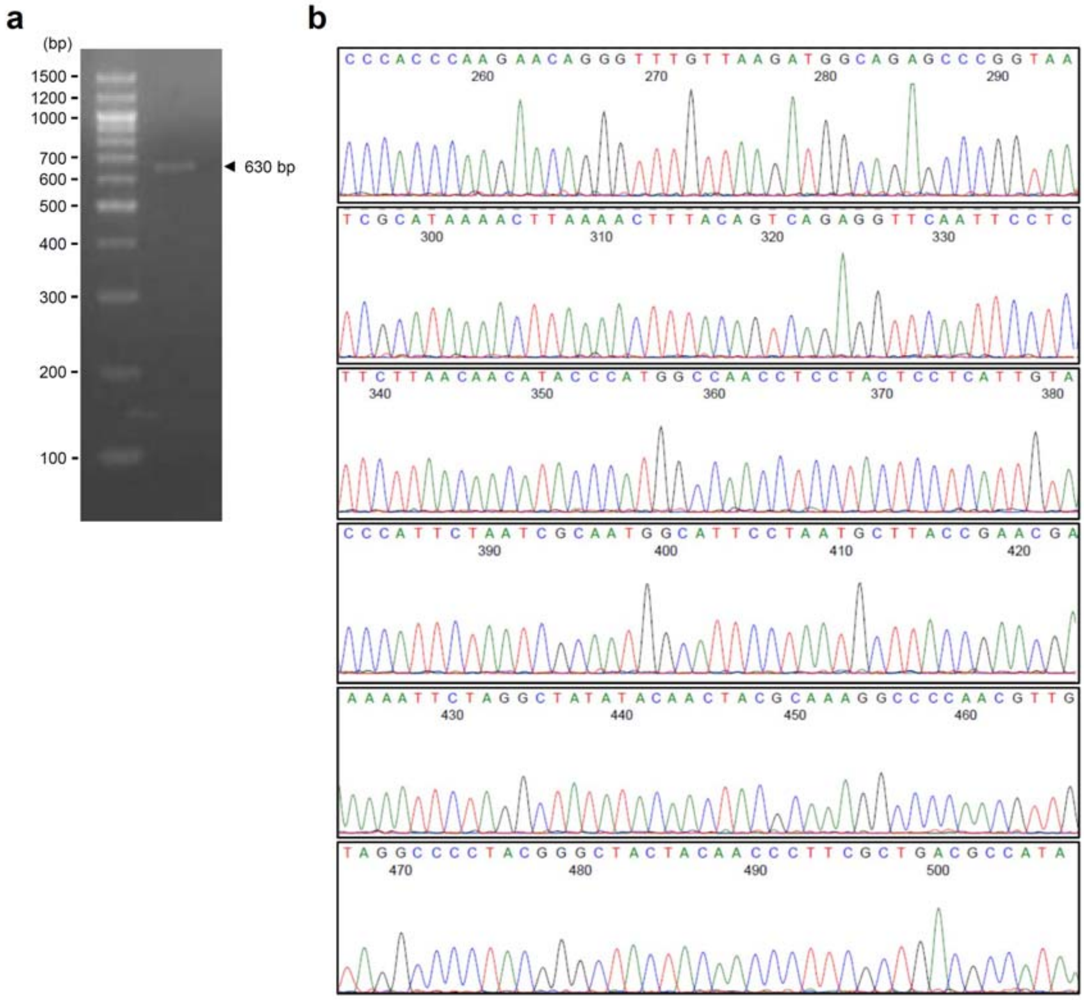

Supplementary Figure 16

The NS1 protein of influenza virus specifically associates with mtDNA.

a,b, HEK293FT cells were transfected with the expression plasmid encoding Flag-tagged NS1 protein. Twenty-four hours after transfection, the cells were infected with ΔNS1 influenza virus for 24 h. Pure cytosolic fraction prepared from digitonin extracts of influenza virus-infected cells were immunoprecipitated with mouse monoclonal antibody against Flag. DNA was extracted from immunoprecipitated samples using QIAquick Nucleotide Removal kit (QIAGEN). A 630-bp portion of the mtDNA was amplified by PCR using human mtDNA-specific primers (forward, 5'-cctagggataacagcgcaat-3', and reverse, 5'-tagaagagcgatggtgagag-3') (**a**), purified (QIAGEN), and sequenced (**b**).

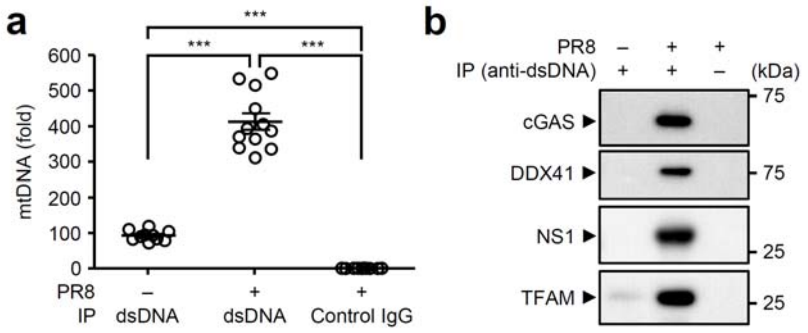

Supplementary Figure 17

Cytosolic mtDNA associates with cGAS, DDX41, NS1, and TFAM.

a,b, cGAS-293FT cells were infected with WT A/PR8 influenza virus for 24 h. Pure cytosolic fraction prepared from digitonin extracts of mock- or influenza virus-infected cGAS-293FT cells were immunoprecipitated with mouse monoclonal antibody against dsDNA (35I9 DNA). DNA was extracted from immunoprecipitated samples using QIAquick Nucleotide Removal kit (QIAGEN). Cytosolic mtDNA was assessed by quantitative PCR (**a**). Immunoprecipitated samples were blotted using the indicated antibodies (**b**). These data are from three independent experiments (**a;** mean ± s.e.m.). ***P < 0.001; (one-way ANOVA and Tukey's test). Source data are provided as a Source Data file.

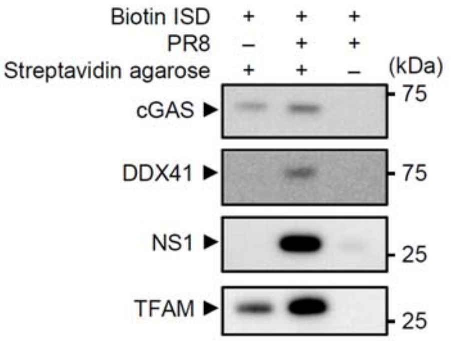

Supplementary Figure 18

Cytosolic ISD associates with cGAS, DDX41, NS1, and TFAM.

cGAS-293FT cells were transfected with biotin-labeled ISD (sense strand sequence: 5'-TACAGATCTACTAGTGATCTAT GACTGATCTGTACATGATCTACA-3'). Nine hours post transfection, cells were infected with WT A/PR8 influenza virus for 15 h. Cell lysates were immunoprecipitated with streptavidin agarose. Immunoprecipitated samples were blotted using the indicated antibodies. Data was representative of three independent experiments.

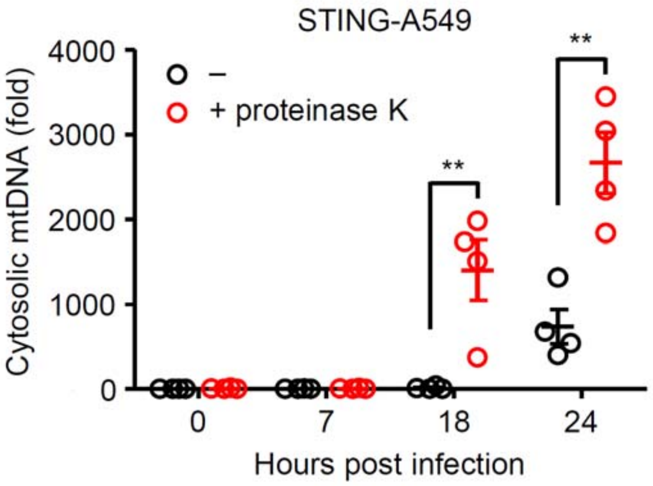

Supplementary Figure 19

Treatment of pure cytosolic extracts of influenza virus-infected cells with proteinase K enhances detectable levels of cytosolic mtDNA.

STING-A549 cells were infected with PR8 virus. At indicated time points, pure cytosolic fraction prepared from digitonin extracts of mock- or influenza virus-infected STING-A549 cells were treated with proteinase K. DNA was extracted from proteinase K-treated pure cytosolic fraction using QIAquick Nucleotide Removal kit (QIAGEN). Cytosolic mtDNA was assessed by quantitative PCR. Data are from three independent experiments (mean ± s.e.m.). **$P < 0.01$; (one-way ANOVA and Tukey's test). Source data are provided as a Source Data file.

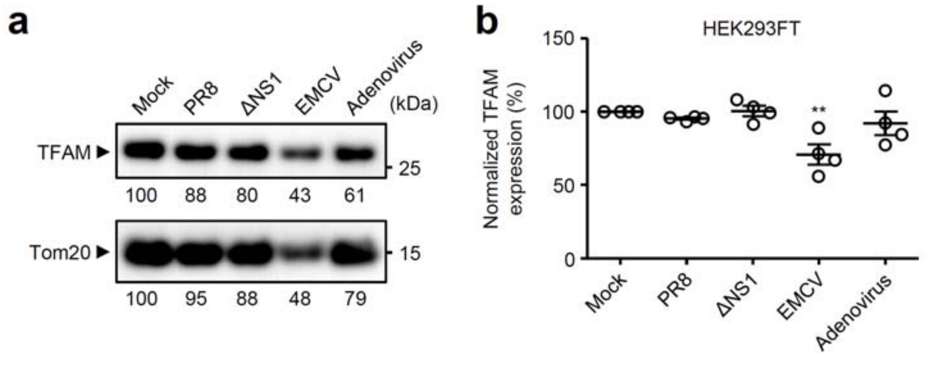

Supplementary Figure 20

EMCV but not influenza virus or adenovirus reduces the expression levels of TFAM.

a,b, HEK293FT cells were infected with WT A/PR8, ΔNS1influenza virus, EMCV, or adenovirus. At 24 h post infection, cells were lysed in 1×TNT buffer, followed by immunoblotting with rabbit monoclonal antibody against TFAM (D5C8) or rabbit polyclonal antibody against Tom20 (FL-145). Indicated below bands are the signal intensities. The value in mock-infected cells was set to 100% (**a**). Expression levels based on signal intensity of TFAM normalized to that of the corresponding Tom20 are shown (**b**). Data are from four independent experiments (**b;** mean ± s.e.m.). $^{**}P < 0.01$; (one-way ANOVA and Tukey's test). Source data are provided as a Source Data file.

Supplementary Table 1

Pre-designed siRNA ID# (Ambion)

Name	siRNA ID or catalog #
Human Bax #1	s1888
Human Bax #2	s1889
Human MAVS #1	s33178
Human MAVS #2	s33179
Human MAVS #3	s33180
Human DDX41 #1	s28120
Human DDX41 #2	s28121
Human DDX41 #3	s224203
Human STING #1	s50644
Human STING #2	s50645
Human STING #3	s226307
Human TFAM #1	s14000
Human TFAM #2	s14001
Human TFAM #3	s14002
Human CX43 #1	s531800
Human CX43 #2	s531801
Negative Control #1	Cat#4390843
Negative Control #2	Cat#4390846

Supplementary Table 2
Oligonucleotides used in generation of recombinant influenza viruses

Name	Sequence (5' to 3')
rgPR8/ΔNS1 F	gacatactgctgaggatgtca
rgPR8/ΔNS1 R	ctgaaagcttgacacagtgtt
rgPR8/M2del$_{29\text{-}31}$ F	atcattgggatcttgcactt
rgPR8/M2del$_{29\text{-}31}$ R	aatagcgagaggatcacttg
rgPR8/M2$_{N31S}$ F	ttgccgcaagtatcattggg
rgPR8/M2$_{N31S}$ R	tagtgagaggatcacttgaa
rgPR8/NS1$_{38/41A}$ F	taagaggaaggggcagcact
rgPR8/NS1$_{38/41A}$ R	gggatgcctgatctgcgcgaagccgatcaaggaatg

Supplementary Table 3

Oligonucleotides used in gene knockout

Name	Sequence (5' to 3')
Human MAVS sense	CACCGcccatcaactcaacccgtgc
Human MAVS anti-sense	AAACgcacgggttgagttgatgggC
Human STING sense	CACCGaagggcgggccgaccgcatt
Human STING anti-sense	AAACaatgcggtcggcccgcccttC
Human DDX41 sense	CACCGcaaatccatgaggcgccccg
Human DDX41 anti-sense	AAACcggggcgcctcatgg atttgC
Human TRIM32 sense #1	CACCGgaggagcggcgtcgggactt
Human TRIM32 anti-sense #1	AAACaagtcccgacgccgctcctcC
Human TRIM32 sense #2	CACCGaactcgtctgcgggaactta
Human TRIM32 anti-sense #2	AAACtaagttcccgcagacgagttC

Supplementary Table 4
Oligonucleotides used in qPCR

Name	Sequence (5' to 3')
Human mtDNA F	cctagggataacagcgcaat
Human mtDNA R	tagaagagcgatggtgagag
Human IFN-β F	ctcctggctaatgtctatca
Human IFN-β R	gcagaatcctcccataatat
Human β-actin F	ctggaacggtgaaggtgaca
Human β-actin R	aagggacttcctgtaacaatgca
Mouse mtDNA F	gccccagatatagcattccc
Mouse mtDNA R	gttcatcctgttcctgctcc
Mouse IFN-β F	gcactgggtggaatgagactattg
Mouse IFN-β R	ttctgaggcatcaactgacaggtc
Mouse GAPDH F	accacagtccatgccatca
Mouse GAPDH R	tccaccaccctgttgctgta
Influenza virus NP F	agaacatctgacatgaggac
Influenza virus NP R	gtcaaaggaaggcacgatc
